# VANGL1 links angiogenesis–stemness programs and tumor microenvironment remodeling: a pan-cancer, multi-omics study with translational validation

**DOI:** 10.3389/fonc.2026.1836811

**Published:** 2026-05-29

**Authors:** Wei Zhou, Lizhen Wang, Mengxing Li, Xuan Liu, Qing Fang, Yang Yu, Songjie Zhang, Jing Guo, Rui Chang, Cheng Zhang, Tongliang Zhou, Yang Liu, Xiaoxia Ren, Honglin Liu, Lihong Liu

**Affiliations:** 1 Department of Pharmacy, China-Japan Friendship Hospital, Beijing, China; 2 Organoid and Regenerative Medicine Center, China-Japan Friendship Hospital, Beijing, China; 3 Beijing Key Laboratory of Critical Bridging Technologies for Chronic Disease Drug Development, Beijing, China; 4 Department of Pathology, Wannan Medical College, Wuhu, Anhui, China; 5 Department of Pathology, the First Affiliated Hospital/Yijishan Hospital, Wannan Medical College, Wuhu, China; 6 Department of Hematology, Affiliated Hospital of Guizhou Medical University, Guiyang, Guizhou, China; 7 Institute of Clinical Medical Sciences, China-Japan Friendship Hospital, Beijing, China; 8 Institute of Respiratory Medicine, Chinese Academy of Medical Sciences, Beijing, China; 9 National Center for Respiratory Medicine, Beijing, China; 10 National Clinical Research Center for Respiratory Diseases, Beijing, China

**Keywords:** angiogenesis, pan-cancer, prognostic biomarker, stemness, tumor microenvironment, VANGL1

## Abstract

**Background:**

The tumor microenvironment (TME) critically determines cancer progression, yet master regulators orchestrating microenvironmental remodeling across malignancies remain elusive. VANGL1, a core planar cell polarity component, has been implicated in tissue morphogenesis, but its pan-cancer landscape and TME regulatory mechanisms are unexplored.

**Methods:**

We conducted comprehensive pan-cancer analysis of VANGL1 across 33 tumor types using bulk transcriptomic, single-cell RNA sequencing, and spatial transcriptomic datasets. Intercellular communication was dissected using CellChat. Functional mechanisms were investigated through PPI networks, CancerSEA, and GSEA. Experimental validation employed loss-of-function studies in PC9, HCT116, and Bel7402 cells, patient-derived tumor organoids, and hepatocellular carcinoma stem cell-derived xenografts.

**Results:**

VANGL1 was significantly upregulated across solid tumors and as-sociated with poor prognosis. Single-cell analysis revealed VANGL1^+^ malignant cells exhibited enhanced communication with fibroblasts, endothelial cells, and immune cells through MIF, VEGF, and ANGPTL pathways. Mechanistically, VANGL1 integrated Wnt/Notch/mTOR signaling with cancer stemness and angiogenesis. VANGL1 knock-down suppressed proliferation, migration, and invasion *in vitro*, and attenuated tumor growth, angiogenesis, and cancer stem cell maintenance *in vivo*.

**Conclusions:**

Our study identifies VANGL1 as a pan-cancer master regulator integrating cancer stemness, angio-genesis, and microenvironmental remodeling, establishing therapeutic rationale for targeting the VANGL1-driven TME niche.

## 1 Introduction

The tumor microenvironment (TME) has emerged as a critical determinant of cancer progression, therapeutic response, and clinical outcomes (1). Comprising malignant cells, cancer-associated fibroblasts (CAFs), endothelial cells, immune infiltrates, and extracellular matrix components, the TME forms a dynamic ecosystem that supports tumor growth through reciprocal intercellular communications (2–4). Key processes including angiogenesis, immunosuppression, and cancer stem cell maintenance are orchestrated by complex ligand-receptor interactions between tumor cells and their stromal counterparts (5–8). Despite the established importance of TME remodeling in malignancy, the molecular master regulators that integrate these diverse cellular processes across multiple cancer types remain incompletely defined (9, 10). Identifying such nodal regulators holds significant translational potential for developing therapies that disrupt the pro-tumorigenic niche.

VANGL1 (VANGL Planar Cell Polarity Protein 1) is a core component of the planar cell polarity (PCP) pathway, originally identified for its essential roles in embryonic development and tissue morphogenesis (11–13). Through interactions with Dishevelled, Prickle, and Frizzled proteins, VANGL1 establishes polarized cell orientation and coordinates collective cell movements (14). While the PCP pathway has been implicated in cancer cell migration and invasion, the specific contribution of VANGL1 to tumor biology, particularly its potential role in regulating the tumor microenvironment, remains largely unexplored (15, 16). Emerging evidence suggests that developmental signaling pathways are frequently coopted during tumorigenesis to sustain cellular plasticity and aggressive tumor phenotypes, prompting investigation of whether VANGL1 functions beyond its canonical polarity role to influence cancer progression (17, 18).

The identification of pan-cancer biomarkers and therapeutic targets represents a central challenge in precision oncology (19). In this study, we conducted a comprehensive pan-cancer analysis of VANGL1 to investigate its expression landscape, clinical relevance, and mechanistic role in tumor progression. We hypothesized that VANGL1 functions as a conserved regulator of tumor-microenvironment crosstalk, integrating cancer stemness, angiogenesis, and stromal remodeling across diverse solid malignancies. Through integrated multi-omics analyses spanning single-cell transcriptomics, spatial transcriptomics, and functional validation, we aimed to establish VANGL1 as a pan-cancer therapeutic target and elucidate the molecular basis of its microenvironmental regulatory functions.

## 2 Materials and methods

### 2.1 Data acquisition and preprocessing

Transcriptomic data and corresponding clinical information were obtained from The Cancer Genome Atlas (TCGA) and the Genotype-Tissue Expression (GTEx) project. Normal tissue TPM expression values from GTEx were paired with tumor TPM expression values from TCGA for comparative analysis. Proteomic data were sourced from the Clinical Proteomic Tumor Analysis Consortium (CPTAC) database.

### 2.2 Survival, clinicopathological, and diagnostic analyses

For survival analysis, patients within each cancer type were stratified into high- and low-VANGL1 expression groups using maximally selected rank statistics (with a mini-mum group proportion of 20%). Kaplan–Meier curves were compared by the log-rank test, and Cox proportional hazards models were applied to estimate hazard ratios (HRs) with 95% confidence intervals (CIs). Given the exploratory pan-cancer scope of the study, reported P values are presented nominally without cross-cancer false discovery rate (FDR) correction. Associations between VANGL1 expression and clinicopathologic features (e.g., AJCC stage or WHO grade) were assessed using the Wilcoxon rank-sum test (for two groups) or the Kruskal–Wallis test (for more than two groups). Diagnostic performance was evaluated by generating receiver operating characteristic (ROC) curves with the pROC package (v1.18.0); the area under the curve (AUC) and its 95% CI (DeLong method) are reported as in-sample discovery estimates, representing an upper bound of potential discriminative ability.

### 2.3 Differential gene expression and functional enrichment analysis

Gene expression data were standardized using Z score normalization. Outliers defined as Z scores less than –3 or greater than 3 were removed prior to downstream analysis. Differential expression between tumor and normal tissues in the gastrointestinal cancer cohort was assessed using the Wilcoxon rank sum test. Significantly differentially expressed genes (DEGs) were further identified with the limma R package.

Functional enrichment analysis was performed using the clusterProfiler R package. Gene Ontology (GO) and Kyoto Encyclopedia of Genes and Genomes (KEGG) pathway enrichments were evaluated for the DEGs. Gene Set Enrichment Analysis (GSEA) was also conducted with clusterProfiler using the Hallmark and KEGG gene sets. Pathway significance was assessed based on the normalized enrichment score (NES) and adjusted P value.

Functional state gene sets corresponding to 14 distinct tumor cell phenotypes were retrieved from the CancerSEA database. Gene Set Variation Analysis (GSVA) was implemented via the R package GSVA using the z−score method to compute enrichment scores for each functional state across samples. Associations between VANGL1 expression and each functional state were then evaluated using Pearson correlation analysis.

### 2.4 Multi-omics regulatory analyses

Spatial transcriptomic analysis. Spatial transcriptomic data were obtained from the Sparkle database (https://grswsci.top/). A pan −cancer spatial transcriptomic atlas was constructed based on gene expression profiles, followed by standardized preprocessing and cell−type annotation. VANGL1 expression was visualized using the SpatialFeaturePlot function from the Seurat package. Tissue regions were then categorized according to the proportion of malignant cells within each spot. Differential expression of VANGL1 between region groups was assessed using the Wilcoxon rank−sum test implemented via the wilcox.test function in R.

Single-cell analysis. Single−cell RNA−sequencing data were acquired from the TISCH2 database. Cell types within the dataset were annotated based on canonical gene expression markers. Dimensionality reduction and visualization were performed using Uniform Manifold Approximation and Projection (UMAP). Cell–cell communication networks were subsequently inferred with the CellChat R package.

Copy number. GSCA provided per-cancer Spearman correlations between GISTIC2.0 copy number and VANGL1 mRNA; BH-FDR q values are reported when available, other-wise nominal P values.

DNA methylation. Correlations between 450K β values (CpGs within ±2 kb of the VANGL1 TSS) and mRNA were summarized per cancer by the strongest inverse probe–expression pair and its q (or P) value.

Epitranscriptomic regulators. Using SangerBox on the TCGA–TARGET–GTEx matrix, we computed Spearman correlations between VANGL1 and 44 writers/readers/erasers (m¹A/m^5^C/m^6^A) in primary tumors; platform-provided BH-FDR was applied when available.

### 2.5 Angiogenesis and stemness associations

Angiogenesis signature. Per-sample angiogenesis scores were derived from WikiPathways WP1539 (24 genes) using ssGSEA (GSVA v1.46.0; method = “ssgsea”); Pearson correlations with VANGL1 were computed per cancer.

Immunofluorescence co-localization. Dual IF for VANGL1 and ALDH1 on FFPE sections was performed as below with DAPI counterstain. Representative images from lung, gastric, colorectal, and ovarian cancers are shown.

TMA IHC quantification. VANGL1 immunostaining in the pan-cancer TMA cohort was quantified using QuPath. Tumor and matched adjacent normal cores were manually annotated, and staining-positive area, mean staining intensity, and QuPath-derived positive staining scores were extracted under identical threshold settings for all samples. Quantitative scores were compared between tumor and matched adjacent normal tissues using two-sided Wilcoxon tests.

Tumor microenvironment deconvolution. TIMER 2.0 modules (MCP-counter, EPIC, xCell, CIBERSORT, CIBERSORT-ABS, quanTIseq) estimated immune/stromal content from bulk RNA-seq. For each cancer, we calculated purity-adjusted partial Spearman correlations (purity from TIMER’s ESTI-MATE-based metric) between VANGL1 and inferred CAFs, endothelial cells (ECs), CD8^+^ T cells, and NK cells. Heatmaps display coefficients (red positive; blue negative); filled squares denote P ≤ 0.05; hatched squares P > 0.05. Sample sizes are annotated on the y-axes.

### 2.6 Experimental models

Cell lines and culture. colorectal adenocarcinoma (HCT116, RRID: CVCL_0291), lung adenocarcinoma (PC9, RRID: CVCL_B260) and hepatocellular carcinoma (Bel7402, RRID: CVCL_5492) were all obtained from Wuhan Pricella Biotechnology Co., Ltd. and respectively cultured in Dulbecco’s Modified Eagle Medium (DMEM) or RPMI-1640 supplemented with 10% fetal bovine serum under standard conditions.

siRNA transfection. Cells at ~30% confluence were transfected with VANGL1 siRNA (sense 5′-GGAGCACAGCAUAUCCCAATT-3′; antisense 5′-UUGGGAUAUGCUGUGCUCCUU-3′) or scrambled control (Shanghai GenePharma) using Lipofectamine RNAiMAX. Knockdown was verified by Western blot at 48 h.

Western blot. Whole-cell lysates were resolved by SDS–PAGE and transferred to PVDF. Primary antibodies: anti-VANGL1 (Proteintech, 14696-1-AP, 1:1000) and anti-GAPDH (Proteintech, 60004-1-Ig, 1:5000). Secondary antibodies: anti-rabbit IgG-HRP (Sigma, A0545, 1:20, 000) and anti-mouse IgG-HRP (GeneTex, GTX213111-01, 1:20, 000). Signals were developed by ECL and imaged on a ChemiDoc system.

Functional assays. Proliferation was measured at 0/24/72/120 h using CCK-8 (Dojin-do). Migration/invasion used 24-well Transwells (8-µm; Corning) with Matrigel coating for invasion. After 24–48 h, cells were fixed and stained with crystal violet. Apoptosis was quantified by Annexin V–FITC/PI and analyzed by flow cytometry (CytoFLEX LX; ≥10, 000 events/sample). Each condition included ≥3 biological replicates.

Tube Formation Assay. Matrigel was coated onto 24-well plates and allowed to solidify at 37 °C for 12h. A1 cells stably expressing shVANGL1 or control construct (con) were seeded at a density of 5, 000 cells per well and incubated at 37 °C with 5% CO_2_ for 48 hours. Tube formation was visualized by fluorescence microscopy and quantified using ImageJ with the Angiogenesis Analyzer plugin.

Sphere Formation Assay. Cells were seeded at a density of 200 cells per well in ultra-low attachment 96-well plates in serum-free DMEM. Spheres were allowed to form at 37 °C with 5% CO_2_ for 7 days. Sphere number and diameter were quantified under light microscopy from three independent experiments.

Xenograft model and immunofluorescence. T3A-A1 hepatic cancer stem cells stably expressing shVANGL1 or shControl (lentiviral transduction; puromycin 2 µg/mL × 14 days; knockdown verified by Western blot) were used for subcutaneous xenografts. Female NCG mice (6–8 weeks, 18–22 g; Beijing Biocytogen) were housed under SPF conditions. Sex selection: females were chosen to reduce co-housing aggression. Cells (5 × 10^5^ in 100 µL PBS) were injected into the right flank (n = 5 per group). Tumor volumes were measured every 4–6 days (V = length × width²/2). On day 22, mice were euthanized; tumors were weighed and processed for protein or histology.

Immunofluorescence. FFPE sections (4 µm) underwent citrate retrieval (10 mM, pH 6.0, 121 °C, 15 min), permeabilization (0.3% Triton X-100), and BSA blocking. Primary antibodies: α-SMA (Abcam, ab5694, 1:300), CD31 (Abcam, ab28364, 1:200), VANGL1 (Proteintech, 14696-1-AP, 1:100), ALDH1 (Proteintech, 60171-1-Ig, 1:200), CD90 (BD, 555593, 1:100). Secondaries: Alexa Fluor 488 (Thermo Fisher, A11034, 1:1000) and Alexa Fluor 594 (Thermo Fisher, A11005, 1:1000); nuclei were counterstained with DAPI (1 µg/mL). Images were acquired at 400× with constant exposure per staining set. Microvessel density (MVD) was quantified on CD31-stained sections using Weidner criteria by two blinded observers.

### 2.7 Statistical analysis

Analyses used R (4.2). Unless specified, tests were two-sided with α = 0.05.

## 3 Results

### 3.1 VANGL1 is upregulated and predicts poor prognosis pan-cancer

**Figure 1:**
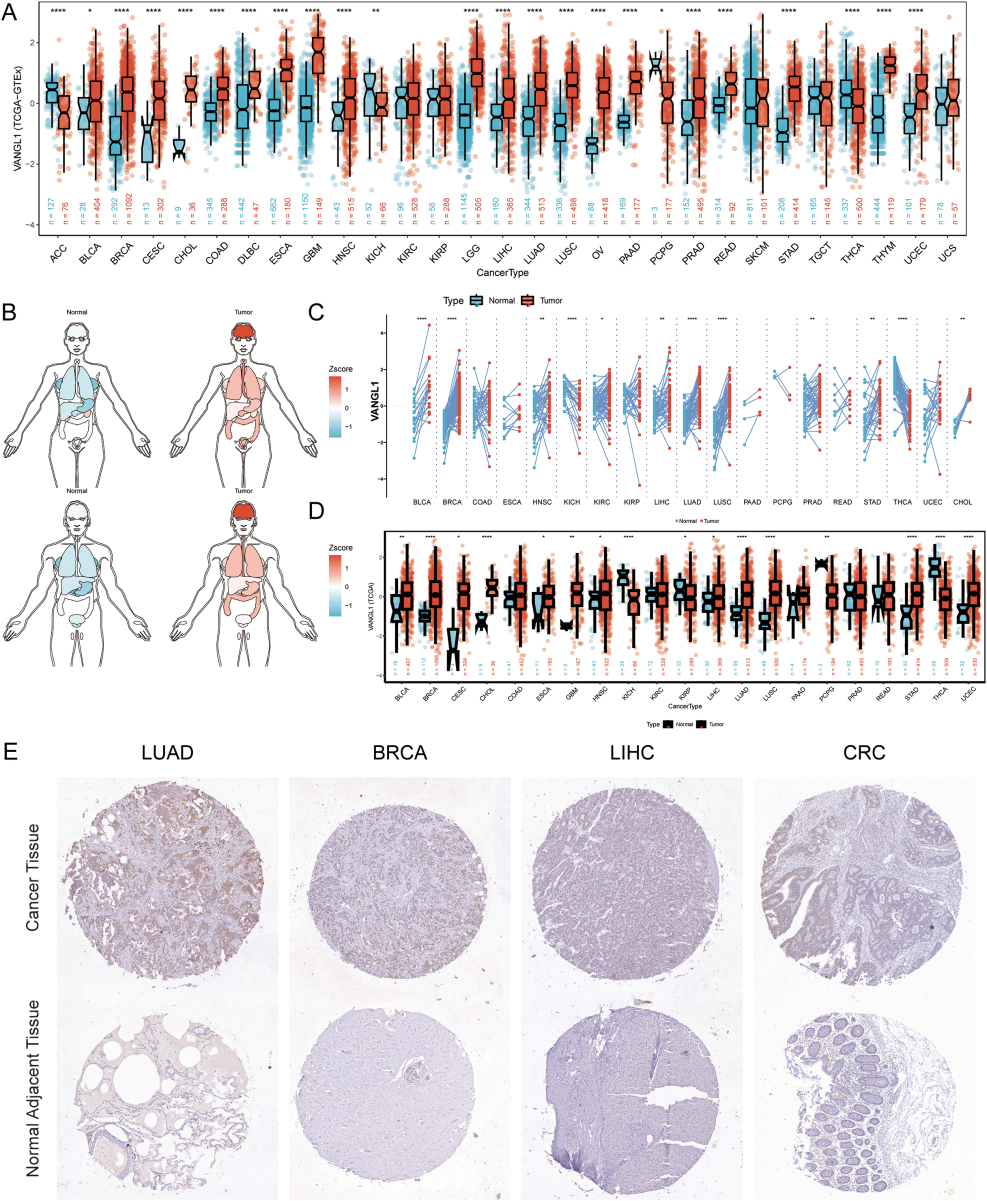
Pan-cancer analysis reveals frequent upregulation of VANGL1 in human tumors. **(A)** VANGL1 mRNA (log_2_ TPM) in 33 TCGA and GTEx cancer types vs. normal tissues. **(B)** Median Z−score of VANGL1 expression in tumors versus matched normal tissues across cancer types. **(C)** Paired analysis of VANGL1 expression in tumors and matched normal tissues within TCGA. **(D)** Expression levels of VANGL1 in tumor and normal tissues from TCGA. **(E)** Matched TMA analysis of VANGL1 protein expression in tumor versus adjacent normal tissues from 42 patients, including LUAD (n = 12), BRCA (n = 12), LIHC (n = 6), and CRC (n = 12). VANGL1 expression was quantified using QuPath-based positive staining scores. Two-sided Wilcoxon tests were performed for each cancer type. *P < 0.05, **P < 0.01, ****P < 0.0001, indicating statistical significance.

VANGL1 was dysregulated in 17 of 33 TCGA tumor types (normals supplemented from GTEx). Expression increased in 13 cancers—including Breast invasive carcinoma (BRCA), Liver hepatocellular carcinoma (LIHC), Lung adenocarcinoma (LUAD), Lung squamous cell carcinoma (LUSC), Stomach adenocarcinoma (STAD), Brain lower grade glioma (LGG), Skin cutaneous melanoma (SKCM), Thymoma (THYM), and Ovarian serous cystadenocarcinoma (OV)—and decreased in 4, notably Thyroid carcinoma (THCA) and Kidney chromophobe (KICH) (**Figures 1A–D**; per-cancer Wilcoxon P values as reported by portals.

Logistic regression analysis implemented with the glm function in R indicated that elevated VANGL1 expression is associated with an increased risk of tumorigenesis (**Supplementary Figure 1A**). Furthermore, receiver operating characteristic (ROC) analysis demonstrated that VANGL1 exhibits strong diagnostic ability in discriminating tumor from normal tissues, supporting its potential as a diagnostic biomarker (**Supplementary Figure 1B**).

These transcriptional findings were further validated at the protein level. Protein expression data from the Clinical Proteomic Tumor Analysis Consortium (CPTAC) (**Supplementary Figures 1C, D**) and paired tissue microarrays (TMA) consistently confirmed higher VANGL1 protein levels in tumor tissues compared with adjacent normal tissues (**Figure 1E**; **Supplementary Figure 1E**).

### 3.2 Survival associations, clinicopathologic correlates, and diagnostic performance of VANGL1

**Figure 2:**
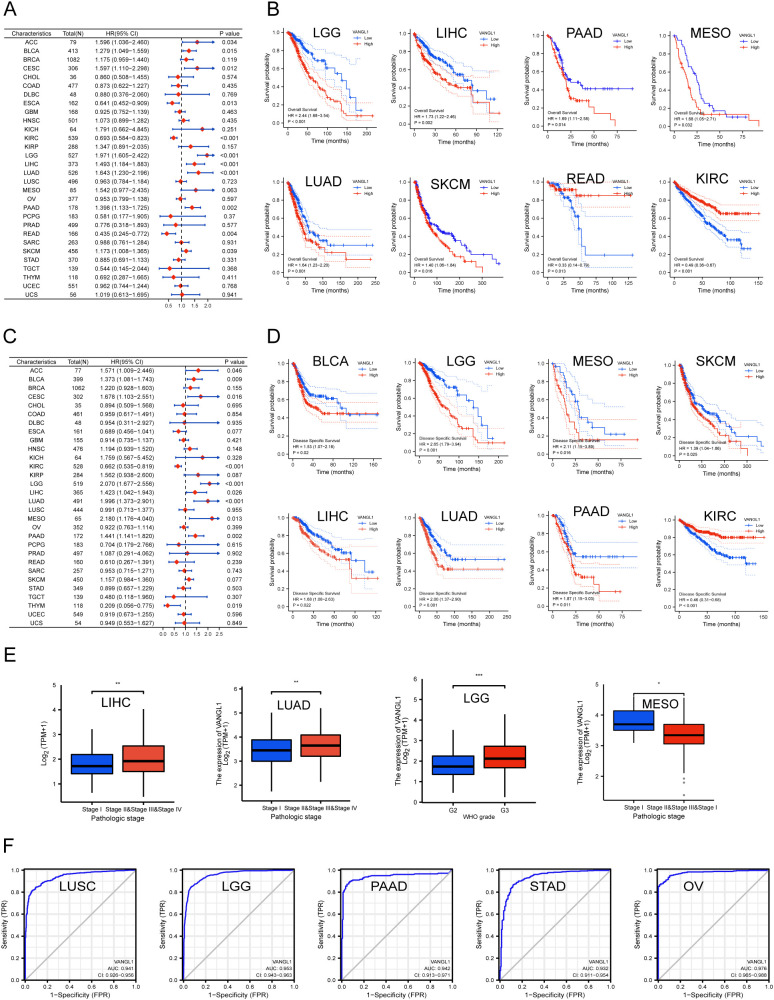
Survival, clinicopathologic correlates, and discovery ROC. **(A)** Univariable Cox HRs (95% CIs) for OS comparing high vs. low VANGL1 (cut-point by maximally selected rank statistics; minimum group proportion 20%). **(B)** Overall-survival Kaplan–Meier curves (LGG, LIHC, PAAD, MESO, LUAD, SKCM, READ, KIRC); log-rank P shown. **(C, D)** Disease-specific survival: Cox HRs and representative Kaplan–Meier curves (BLCA, LGG, MESO, SKCM, LIHC, LUAD, PAAD, KIRC). **(E)** VANGL1 vs. stage/grade in LIHC, LUAD, LGG, MESO (Wilcoxon or Kruskal–Wallis; *P<0.05, **P<0.01, ***P<0.001, ****P<0.0001). **(F)** In-sample ROC for tumor–normal classification in LUSC, LGG, PAAD, STAD, OV; AUC with DeLong 95% CI.

Higher VANGL1 associated with worse OS in multiple cancers (**Figure 2A**): LGG HR 1.97 (95% CI 1.61–2.42), LIHC 1.49 (1.18–1.88), LUAD 1.64 (1.23–2.20; all P<0.001). Kaplan–Meier curves showed concordant separations (**Figure 2B**), and DSS analyses yielded similar patterns (**Figures 2C, D**). VANGL1 rose with pathologic stage in LIHC and LUAD and with WHO grade in LGG, whereas Mesothelioma (MESO) showed an opposite trend (**Figure 2E**; Wilcoxon/Kruskal–Wallis, nominal P values). In-sample ROC analyses indicated tumor–normal discrimination in selected cancers, with AUCs of ~0.98 in OV, ~0.95 in LGG, and ~0.93 in STAD (**Figure 2F**); these are discovery-stage upper-bound estimates pending ex-ternal validation.

Integrative multi-omics analysis revealed that VANGL1 overexpression is governed by genomic, epigenetic, and post-transcriptional mechanisms. Copy number gains positively correlated with VANGL1 mRNA in READ, UVM, LUSC, and LUAD (**Supplementary Figure 2A**). Promoter-proximal hypomethylation accompanied higher VANGL1 expression in LGG/GBM, BRCA, LUSC, and LUAD (**Supplementary Figure 2B**). Additionally, VANGL1 co-varied with m^6^A regulators (METTL3/METTL14, FTO, YTHDF1/2/3) across cancers (**Supplementary Figure 2C**), supporting a multilayer model of VANGL1 regulation.

### 3.3 Multi-omics correlates of VANGL1 dysregulation

**Figure 3:**
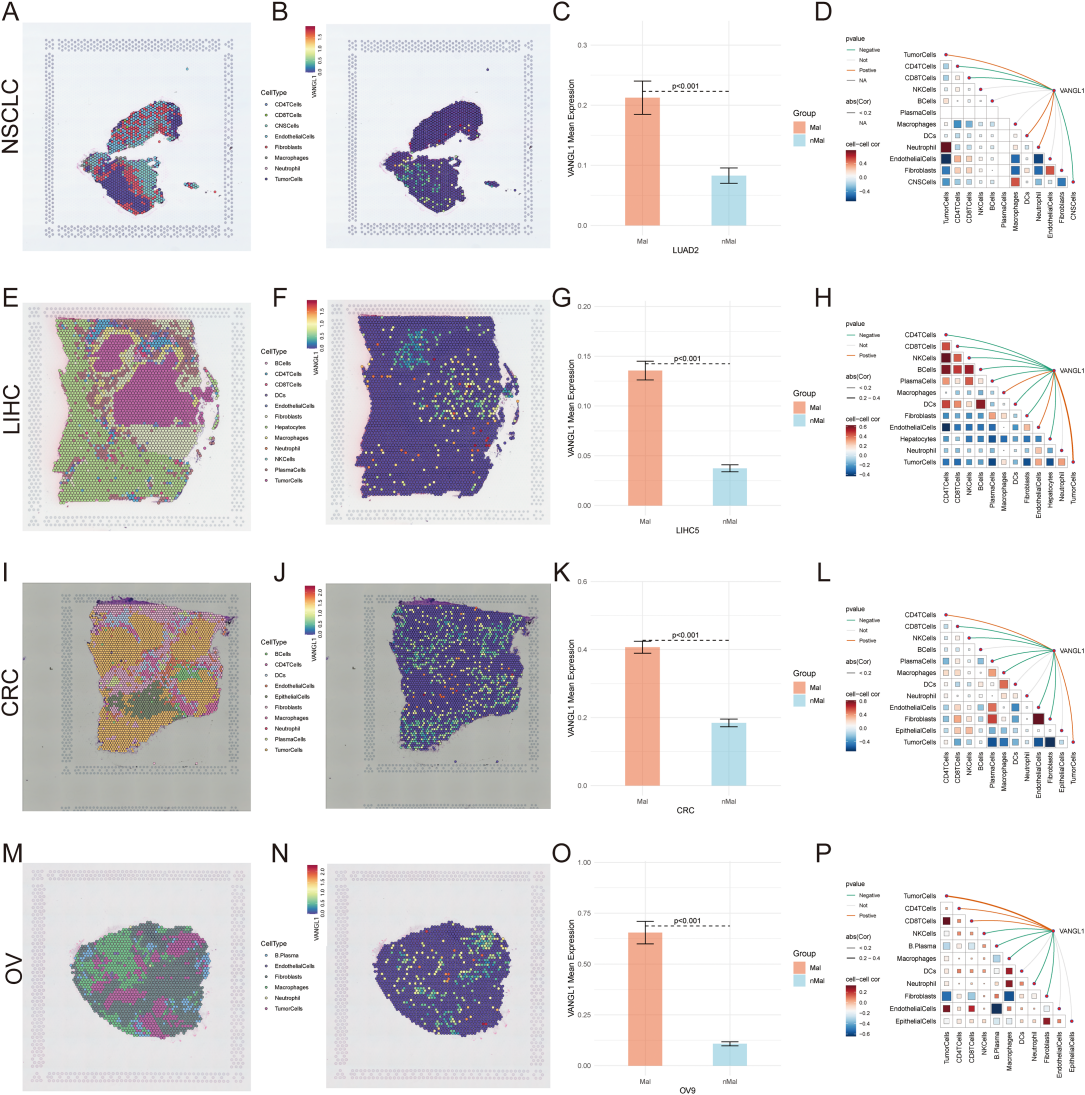
Spatial transcriptomics reveals VANGL1 enrichment in malignant cells and association with tumor microenvironment remodeling. **(A–D)** Non-small cell lung cancer (NSCLC) spatial transcriptomics analysis. **(A)** Cell type annotation on tissue section. **(B)** VANGL1 expression distribution. **(C)** Quantification of VANGL1 mean expression in malignant (Mal) versus non-malignant (nMal) cells. **(D)** Cell-cell communication circle plot showing VANGL1-associated interactions between malignant cells and microenvironment components. **(E–H)** Hepatocellular carcinoma (LIHC) spatial analysis. **(E)** Cell type annotation. **(F)** VANGL1 expression distribution. **(G)** VANGL1 expression quantification in malignant versus non-malignant cells. **(H)** Communication network highlighting VANGL1-mediated crosstalk. **(I–L)** Colorectal cancer (CRC) spatial analysis. **(I)** Cell type annotation. **(J)** VANGL1 expression distribution. **(K)** VANGL1 expression quantification in malignant versus non-malignant cells. **(L)** Cell-cell communication analysis of VANGL1-associated interactions. **(M–P)** Ovarian cancer (OV) spatial analysis. **(M)** Cell type an-notation. **(N)** VANGL1 expression distribution. **(O)** VANGL1 expression quantification in malignant versus non-malignant cells. **(P)** Communication network showing VANGL1-driven microenvironment interactions.

To further elucidate the cellular distribution of VANGL1 within tumor tissues, we employed spatially resolved transcriptomics. In NSCLC (**Figures 3A, B**), LIHC(**Figures 3E, F**), CRC(**Figures 3I, J**), and OV (**Figures 3M, N**), we deconvoluted the major cell type and quantified VANGL1 expression within each tissue spot. The analysis revealed that VANGL1 expression was predominantly elevated in malignant epithelial cells. Furthermore, upon stratifying tissue regions based on the proportion of malignant cells, we consistently observed stronger VANGL1 signals in malignant-enriched domains (**Figures 3C, G, K, O**). Cell-cell communication analysis further indicated potential interactions between malignant cells, endothelial cells, and specific immune cell populations, suggesting that VANGL1 may influence multiple functional aspects of the TME (**Figures 3D, H, L, P**).

**Figure 4:**
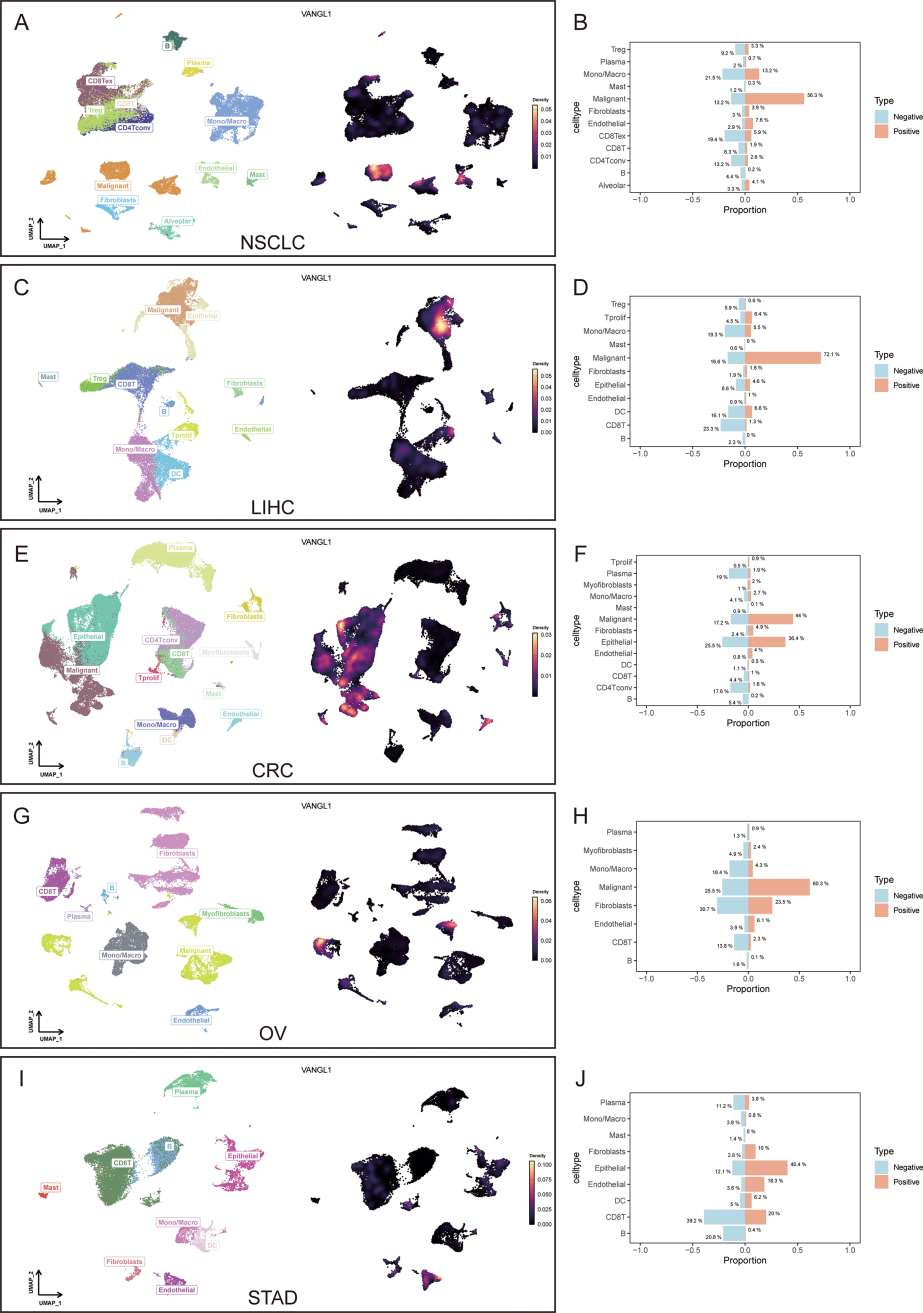
Single-cell atlas of VANGL1 expression across solid malignancies. **(A)** UMAP visualization of cell type annotation (left) and VANGL1 expression distribution (right) in NSCLC. **(B)** Proportion of VANGL1-positive versus VANGL1-negative cells across cell types in NSCLC. **(C)** UMAP visualization of cell type annotation (left) and VANGL1 expression distribution (right) in LIHC. **(D)** Cell type-specific proportion of VANGL1-positive cells in LIHC. **(E)** UMAP visualization of cell type annotation (left) and VANGL1 expression distribution (right) in CRC. **(F)** Proportion analysis of VANGL1 expression across cell populations in CRC. **(G)** UMAP visualization of cell type annotation (left) and VANGL1 expression distribution (right) in OV. **(H)** VANGL1-positive cell distribution across cell types in OV. **(I)** UMAP visualization of cell type annotation (left) and VANGL1 expression distribution (right) in STAD. **(J)** Proportion of VANGL1-positive cells in each cell type in STAD.

To validate the spatial transcriptomic findings, we analyzed single-cell RNA sequencing datasets from multiple tumor types available in the TISCH2 database. Consistent with the spatial profiling results, VANGL1 expression was predominantly localized to malignant epithelial cells across cancers (**Figures 4A, C, E, G**). Comparative analysis further revealed distinct transcriptional profiles between VANGL1−positive and VANGL1−negative malignant cell subpopulations, underscoring the association of VANGL1 expression with specific malignant states (**Figures 4B, D, F, H**). Notably, in the gastric cancer single-cell dataset, we observed that VANGL1 was expressed not only in tumor-derived epithelial cells but was also predominantly enriched in endothelial cells (**Figure 4I**). Furthermore, the proportion of endothelial cells was significantly higher among VANGL1-positive cells compared to VANGL1-negative populations (**Figure 4J**). This finding further suggests a complex, multi-faceted role for VANGL1 in shaping the intratumoral microenvironment.

### 3.4 VANGL1 orchestrates pro-tumorigenic tumor microenvironment remodeling across multiple cancer types

**Figure 5:**
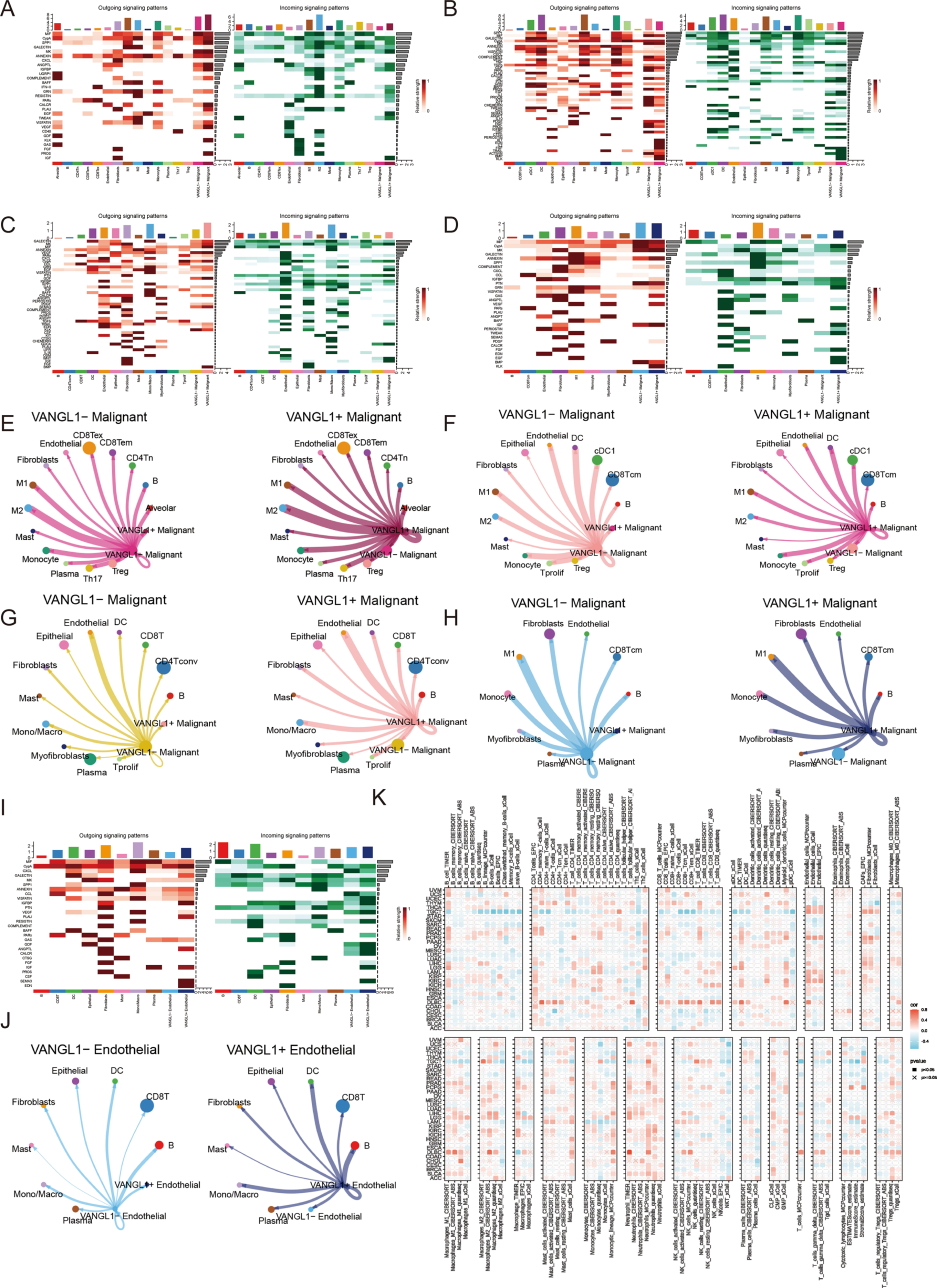
VANGL1 drives tumor microenvironment remodeling across solid malignancies. **(A–D)** Outgoing (red) and incoming (green) signaling heatmaps of VANGL1^−^ and VANGL1^+^ malignant cells in **(A)** LUAD, **(B)** LIHC, **(C)** CRC, and **(D)** OV. VANGL1^+^ cells show upregulated MIF, GALECTIN, SPP1, VEGF, ANGPTL, and EGF signaling. **(E–H)** Cell-cell communication networks of VANGL1^−^ (left) and VANGL1^+^ (right) malignant cells in **(E)** LUAD, **(F)** LIHC, **(G)** CRC, and **(H)** OV. Edge thickness indicates communication strength. VANGL1^+^ cells exhibit intensified crosstalk with immune cells, fibroblasts, and endothelial cells. **(I)** Signaling heatmaps of VANGL1^−^ and VANGL1^+^ malignant cells in STAD. **(J)** Communication networks of VANGL1^−^ and VANGL1^+^ endothelial cells in STAD. VANGL1^+^ endothelial cells show enhanced interactions with malignant cells and immune infiltrates. **(K)** Pan-cancer immune infiltration heatmaps showing correlation between VANGL1 expression and immune cell abundance across tumor types.

To dissect how VANGL1 shapes the TME, we performed CellChat-based intercellular communication analysis across five major solid tumor types. In LUAD, LIHC, CRC, and OV, VANGL1^+^ malignant cells exhibited profoundly altered outgoing signaling patterns compared to VANGL1^−^ counterparts, characterized by upregulated secretion of MIF, GALECTIN, SPP1, VEGF, ANGPTL, and EGF (**Figures 5A–D**). Concurrently, these cells displayed enhanced incoming signals from fibroblasts, endothelial cells, and myeloid populations, indicating bidirectional TME crosstalk.

Network visualization revealed that VANGL1^+^ malignant cells establish extensive and strengthened connections with immune and stromal compartments. In LUAD, VANGL1^+^ malignant cells intensified communications with CD8^+^ T cells, CD4^+^ T cells, B cells, M1/M2 macrophages, and fibroblasts (**Figure 5E**). LIHC showed a distinct stromal-enriched architecture where VANGL1^+^ malignant cells preferentially engaged regulatory T cells (Treg), exhausted CD8^+^ T cells (CD8Tex), and proliferating T cells (Tprolif) (**Figure 5F**).

CRC and OV analyses further highlighted VANGL1-driven stromal remodeling, with VANGL1^+^ malignant cells exhibiting enhanced crosstalk with myofibroblasts, endothelial cells, and monocyte/macrophage lineages (**Figures 5G, H**), suggestive of desmoplastic and pro-angiogenic niche formation.

In STAD, VANGL1^+^ malignant cells similarly upregulated pro-tumorigenic outgoing signals including VEGF, ANGPTL, and TGFβ pathways (**Figure 5I**). Notably, VANGL1^+^ endothelial cells in STAD demonstrated markedly amplified incoming communications from malignant cells and broadened interactions with CD8^+^ T cells, B cells, and monocytes/macrophages compared to VANGL1^−^ endothelial cells (**Figure 5J**), implicating VANGL1 in vascular normalization and immune cell trafficking. Pan-cancer immune deconvolution analysis confirmed that VANGL1 expression correlates with distinct immune infiltration landscapes across tumor types (**Figure 5K**). Collectively, these findings establish VANGL1 as a master regulator integrating angiogenesis, immunosuppression, and stromal activation across diverse solid tumors.

### 3.5 VANGL1 drives functional plasticity and stemness maintenance through polarity and oncogenic signaling

**Figure 6:**
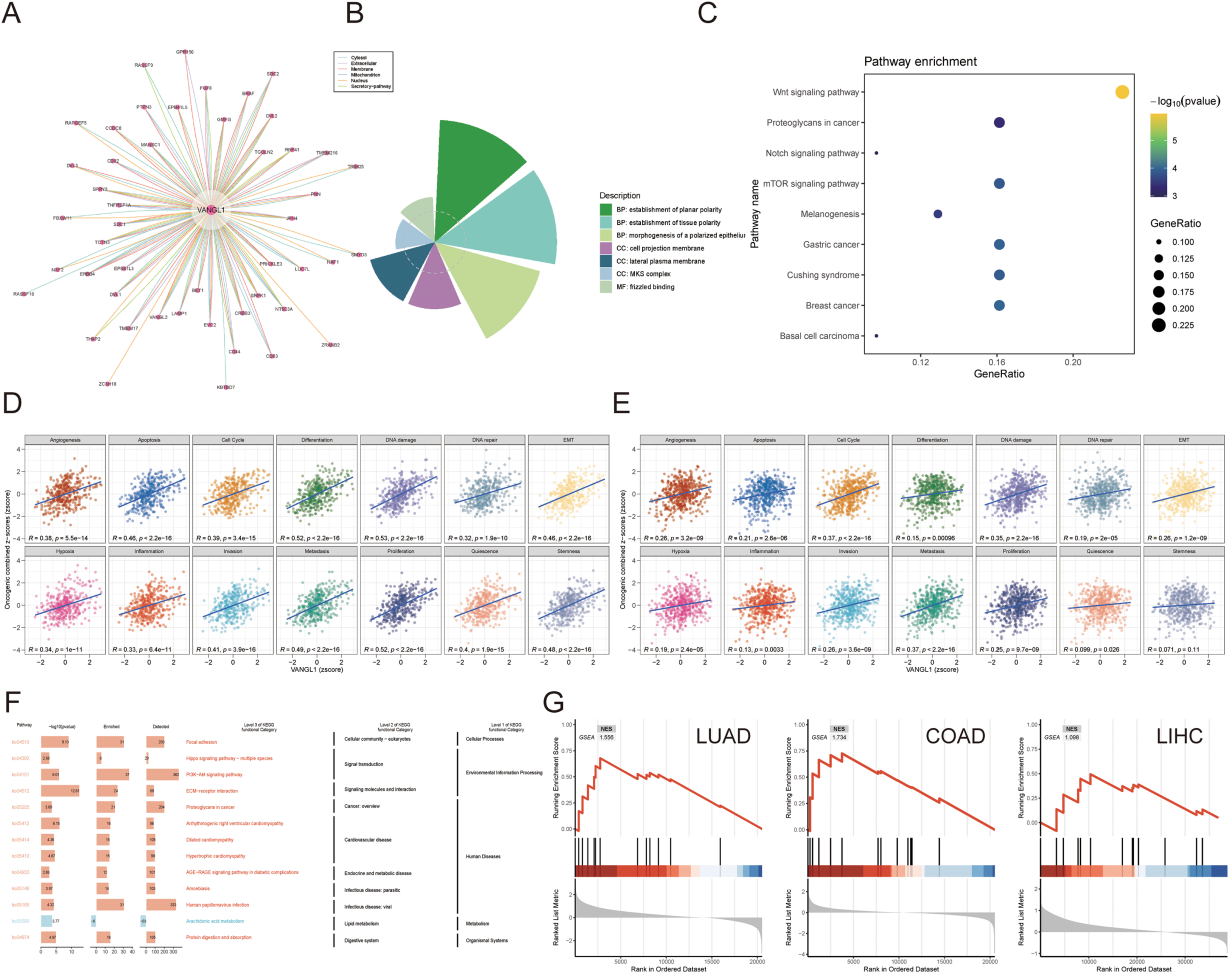
VANGL1 integrates polarity signaling with oncogenic pathways and cancer stemness. **(A)** VANGL1-centered protein-protein interaction network showing 55 interacting partners. **(B)** GO enrichment analysis of VANGL1-associated proteins, highlighting planar cell polarity and tissue polarity. **(C)** KEGG pathway enrichment analysis with Wnt signaling, Notch signaling, and mTOR as top hits. **(D, E)** CancerSEA analysis correlating VANGL1 expression with functional states in **(D)** LIHC and **(E)** LUAD. VANGL1 posi-tively correlates with angiogenesis, cell cycle, EMT, and DNA repair. **(F)** Pan-cancer KEGG functional enrichment of VANGL1-associated genes, showing focal adhesion, PI3K-Akt, and ECM-receptor interaction pathways. **(G)** GSEA of stemness signatures in VANGL1-high versus VANGL1-low tumors. Significant enrichment in LUAD, COAD, and LIHC confirms VANGL1’s role in cancer stem cell maintenance.

To elucidate the molecular mechanisms underlying VANGL1-driven tumor progression, we constructed a VANGL1-centered protein-protein interaction network. GO enrichment analysis revealed predominant enrichment in establishment of planar cell polarity and tissue polarity (**Figures 6A, B**), consistent with VANGL1’s canonical function in non-canonical Wnt signaling. KEGG pathway analysis further highlighted Wnt signaling pathway, proteoglycans in cancer, Notch signaling pathway, and mTOR signaling pathway (**Figure 6C**), linking VANGL1 to core cancer hallmarks including stemness maintenance and metabolic reprogramming.

Using CancerSEA to quantify 14 functional dimensions in cancer cells, we found that VANGL1 expression in LIHC and LUAD significantly correlated with angiogenesis, cell cycle, EMT, and DNA repair (**Figures 6D, E**; **Supplementary Figure S3**). These findings mechanistically connect VANGL1 to the aggressive tumor phenotypes observed in our single-cell analysis, particularly the enhanced angiogenic signaling and stromal remodeling mediated by VANGL1^+^ malignant and endothelial cells.

Pan-cancer KEGG enrichment analysis revealed that VANGL1-associated genes were significantly enriched in focal adhesion, PI3K-Akt signaling, pathways in cancer, and ECM-receptor interaction (**Figure 6F**), underscoring VANGL1’s broad involvement in tumor-microenvironment crosstalk. Crucially, Gene Set Enrichment Analysis demonstrated that high VANGL1 expression was significantly enriched for stemness cell signatures in LUAD, COAD, and LIHC (**Figure 6G**), directly implicating VANGL1 in cancer stem cell maintenance. Collectively, these molecular findings corroborate our single-cell observations and establish VANGL1 as a master regulator integrating planar cell polarity, angiogenesis, and stemness in tumor progression.

### 3.6 VANGL1 associates with angiogenesis and stemness programs

**Figure 7:**
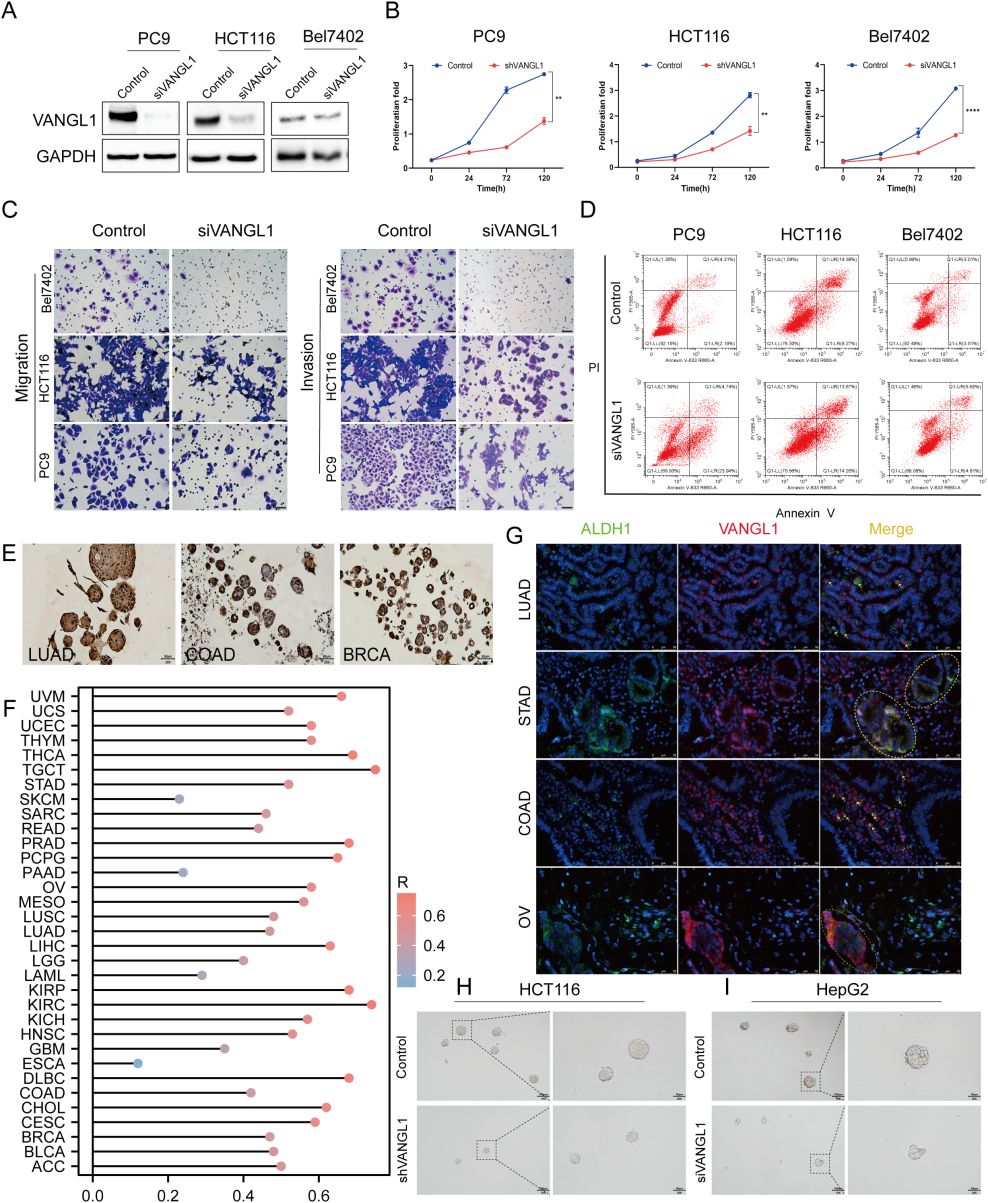
VANGL1 promotes tumor proliferation, metastasis, angiogenesis, and stemness. **(A)** Western blot validation of VANGL1 knockdown efficiency in PC9 (LUAD), HCT116 (CRC), and Bel7402 (LIHC) cell lines. GAPDH serves as loading control. **(B)** Cell proliferation assays showing reduced growth of VANGL1-silenced cells compared to control. Data represent mean ± SD from three independent experiments. *P < 0.05, **P < 0.01, ****P < 0.0001. **(C)** Transwell migration (left) and invasion (right) assays demonstrating attenuated motility of VANGL1-knockdown cells. Representative images shown; quantification from three independent experiments. **(D)** Annexin V/PI flow cytometry analysis showing apoptosis induction following VANGL1 silencing. Data are representative of three independent experiments. **(E)** Representative immunohistochemical staining of VANGL1 in patient-derived tumor organoids from LUAD, COAD, and BRCA, showing heterogeneous expression with enrichment in peripheral regions. **(F)** Pan-cancer correlation between VANGL1 expression and angiogenesis activity. Dot plot shows Pearson correlation coefficients between VANGL1 expression and ssGSEA scores of WP1539 angiogenesis gene set across 33 cancer types. Red indicates positive correlation (R > 0.4). **(G)** Immunofluorescence co-staining of VANGL1 (red) and ALDH1 (green) in patient tumor tissues from LUAD, STAD, COAD, and OV. DAPI (blue) indicates nuclei. Dashed circles highlight VANGL1/ALDH1 co-positive stem-like cell populations. Scale bars indicate 50 μm. **(H)** Representative images of sphere formation assay in HCT116 cells transfected with shVANGL1 or control construct (con). Scale bars, 100 μm and 50 μm. **(I)** Representative images of sphere formation assay in HepG2 cells transfected with siVANGL1 or negative control (siNC). Scale bars, 100 μm and 50 μm.

To functionally validate the pro tumorigenic role of VANGL1 indicated by our bioinformatic analyses, we conducted loss of function studies in three cancer cell lines representing lung adenocarcinoma (PC9), colorectal carcinoma (HCT116), and hepatocellular carcinoma (Bel7402). Efficient knockdown of VANGL1 was confirmed by Western blot analysis (**Figure 7A**). Silencing of VANGL1 significantly suppressed cell proliferation (**Figure 7B**), impaired migratory and invasive capacities, and enhanced apoptosis in all three cell lines (**Figures 7C, D**). These functional findings are consistent with our multi omics observations that VANGL1 positive malignant cells exhibit enhanced interactions with the tumor microenvironment.

To assess VANGL1 expression in patient-derived tumor models, we performed immunohistochemical staining of tumor organoids from LUAD, COAD, and BRCA. VANGL1 showed strong and heterogeneous expression across all three cancer types, with enrichment in peripheral and invasive regions of the organoids (**Figure 7E**), consistent with its role in tumor-microenvironment interaction.

Correlating with our single-cell finding that VANGL1^+^ malignant and endothelial cells exhibit enhanced angiogenic signaling, we analyzed the association between VANGL1 expression and angiogenesis activity across 33 cancer types. Single-sample GSEA (ssGSEA) of the WP1539 angiogenesis gene set revealed significant positive correlations with VANGL1 expression in majority of cancers (**Figure 7F**). This pan-cancer analysis mechanistically links VANGL1 to the angiogenic programs observed in our cell-cell communication analysis.

To further validate VANGL1’s association with cancer stemness at the tissue level, we performed immunofluorescence staining of patient tumor tissues. VANGL1 (red) co-localized with the cancer stem cell marker ALDH1 (green) in LUAD, STAD, COAD, and OV (**Figure 7G**, dashed circles), with VANGL1 predominantly enriched in ALDH1-positive stem-like cell populations. To provide functional evidence for the involvement of VANGL1 in cancer stemness, we performed sphere formation assays in HepG2 and HCT116 cells following VANGL1 knockdown. VANGL1 silencing significantly reduced both sphere number and sphere diameter compared with control cells (**Figures 7H, I**; **Supplementary Figure 4**), supporting an association between VANGL1 and tumor cell self-renewal capacity. These experimental data substantiate our computational predictions and establish VANGL1 as a functional driver of tumor proliferation, metastasis, angiogenesis, and cancer stem cell maintenance.

### 3.7 VANGL1 depletion suppresses tumor growth, angiogenesis, and cancer stemness *in vivo*

**Figure 8:**
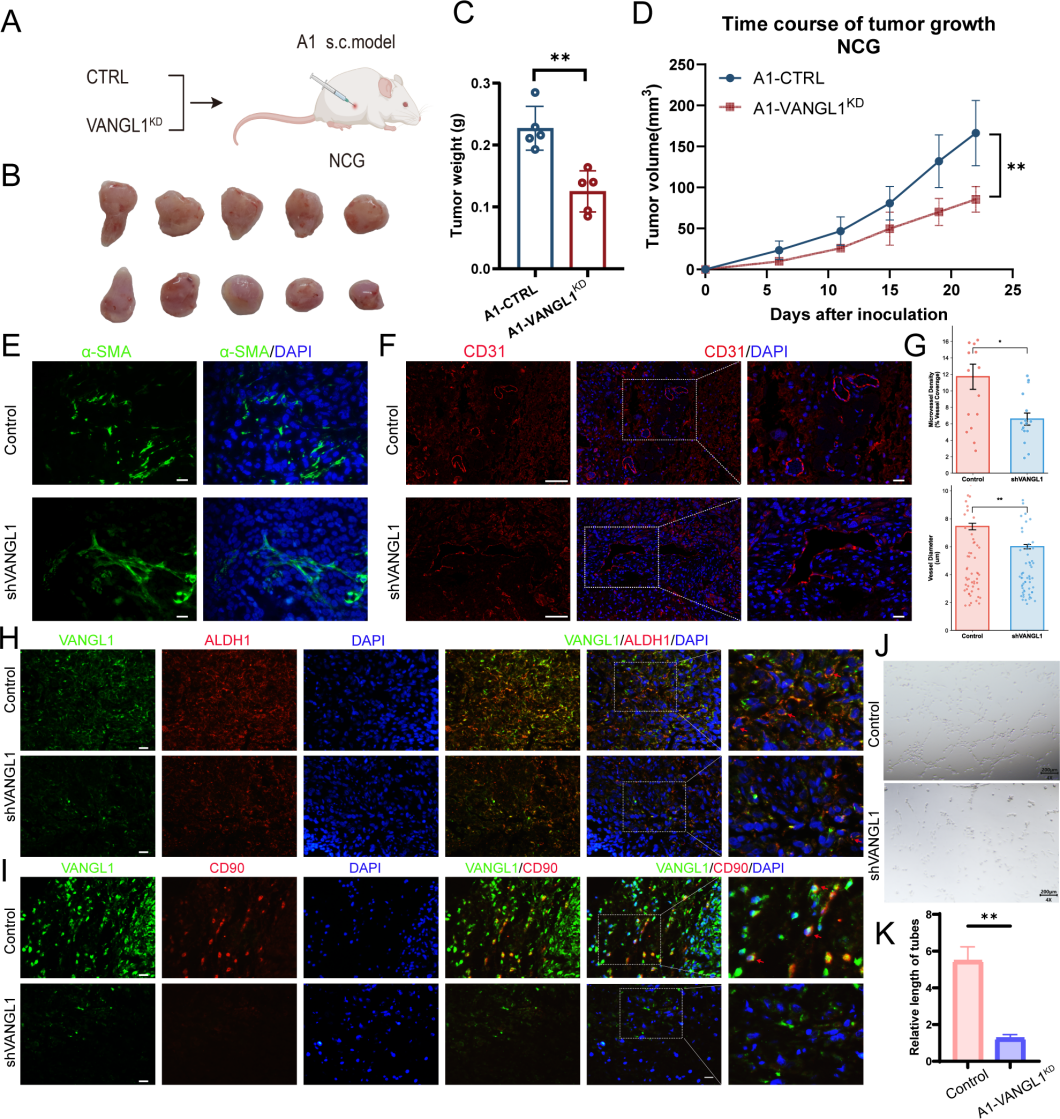
VANGL1 depletion suppresses liver cancer stem cell-driven tumor growth and attenuates vascular/stromal remodeling *in vivo*. **(A)** Schematic of subcutaneous xenograft establishment using T3A-A1 hepatocellular carcinoma stem cells with stable VANGL1 knockdown (VANGL1KD) or control vectors in NCG mice. **(B)** Representative images of excised tumors from T3A-A1-CTRL and T3A-A1-VANGL1KD groups at endpoint (day 21). **(C)** Tumor weights at endpoint. Data represent mean ± SD; n = 5 per group. **(D)** Time course of tumor growth. Data represent mean ± SD; n = 5 per group. **P < 0.01. **(E)** Immunofluorescence staining of α-SMA (green) CAFs showing attenuated stromal signals in knockdown tumors. Scale bars, 5μm. **(F)** Representative immunofluorescence images of CD31 (red). Control tumors exhibit large, irregular vascular lacunae, whereas shVANGL1 tumors display pruned, discrete capillaries, indicating structural normalization. Insets show magnified regions. Scale bars, 5 μm. **(G)** Quantification of microvessel density (MVD, upper panel) and mean vessel diameter (lower panel). VANGL1 knockdown significantly reduced both MVD and vessel caliber. **(H)** Immunofluorescence co-staining of VANGL1 and ALDH1 showing diminished cancer stem cell populations in VANGL1^KD^ tumors. Insets show magnified regions with VANGL1/ALDH1 co-positive cells. Scale bars, 5μm. **(I)** Immunofluorescence co-staining of VANGL1 and CD90 showing reduced mesenchymal/stromal cells in VANGL1KD tumors. Insets show magnified regions with VANGL1/CD90 co-positive cells. Scale bars, 5 μm. **(J–K)** Representative fluorescence microscopy images and quantification of Matrigel-based vascular-like tube formation by A1 hepatocellular carcinoma stem-like cells stably expressing shVANGL1 or control vector. Images were captured 48 h after seeding. Tube-like network formation was quantified using ImageJ Angiogenesis Analyzer. Scale bar, 200 μm. Data are presented as mean ± SD from three independent experiments. *P<0.05, **P < 0.01.

To validate the pro-tumorigenic functions of VANGL1 in an *in vivo* context, we established subcutaneous xenograft models using T3A-A1 cells (hepatocellular carcinoma stem cells) with stable VANGL1 knockdown (VANGL1^KD^) or control vectors in NCG mice (**Figure 8A**). Tumor volume and weight were significantly reduced following VANGL1 knockdown *in vivo*. These findings demonstrate that VANGL1 is essential for cancer stem cell-driven tumorigenesis. WGCNA in hepatocellular carcinoma identified a VANGL1-associated blue module enriched in cell cycle and DNA replication genes, strongly correlated with tumor grade and poor survival (**Supplementary Figure 5**). This module exhibited robust preservation (cor = 0.91, p < 1e-200) and was functionally annotated to TGF-beta signaling and viral carcinogenesis pathways, mechanistically linking VANGL1 to proliferative control and microenvironmental remodeling in LIHC.

To elucidate the mechanisms underlying VANGL1-driven tumor progression, we examined stromal components and cancer stem cell maintenance *in vivo*. Immunofluorescence staining revealed a distinct remodeling of the tumor stroma, characterized by diminished α-SMA expression levels (**Figure 8E**). Crucially, morphological analysis of CD31 staining revealed a transition towards structural normalization: the dilated, irregular vascular lacunae observed in control tumors were replaced by pruned, streamlined capillary-like structures in the knockdown group (**Figure 8F**). This structural normalization was quantitatively confirmed by a significant reduction in mean vessel diameter (**Figure 8G** lower), alongside a concomitant decrease in vessel density (**Figure 8G** upper), which reflects the pruning of aberrant vascular branches. These findings suggest that VANGL1 inhibition disrupts the abnormal stromal support required for maintaining chaotic tumor vasculature. Concurrently, VANGL1 depletion markedly diminished ALDH1^+^ cancer stem cells and CD90^+^ mesenchymal/stromal populations (**Figures 8H, I**), establishing VANGL1 as a critical regulator sustaining the stem cell niche and stromal remodeling in hepatocellular carcinoma. To complement the *in vivo* vascular phenotype, we further assessed whether VANGL1 affects vascular-like network formation by hepatocellular carcinoma stem-like cells. Based on our previous evidence that liver cancer stem cells can acquire endothelial-like features and form vascular-like structures, A1 cells stably expressing shVANGL1 or control vector were seeded onto Matrigel. VANGL1 knockdown markedly reduced vascular-like tube formation compared with control cells, as quantified by ImageJ Angiogenesis Analyzer (**Figures 8J, K**). These data suggest that VANGL1 contributes to angiogenesis-associated tumor cell plasticity, although endothelial-cell-based conditioned-medium assays will be required to directly assess paracrine effects on vascular endothelial cells.

## 4 Discussion

Tumor progression is governed by dynamic interactions between malignant cells and their microenvironment (20, 21). However, the molecular regulators that integrate these processes remain incompletely understood. In this study, we identify VANGL1 as a key coordinator of cancer stemness, angiogenesis, and immune-stromal crosstalk across multiple solid tumors. Through a multi−omics approach and experimental validation, we identified that VANGL1 as a candidate biomarker linked to adverse outcomes and tumor microenvironment remodeling.

VANGL1 is upregulated across multiple tumor types, and its elevated expression correlates with poorer patient survival (22–24). Spatial transcriptomic and single−cell analyses in lung adenocarcinoma, hepatocellular carcinoma, colorectal cancer, ovarian cancer, and gastric cancer further revealed that VANGL1+ malignant cells exhibit a distinct intercellular communication architecture, characterized by enhanced signaling output through pathways such as MIF and VEGF. These molecular signals collectively foster an angiogenic and stroma−activated state associated with immune exclusion, which hallmarks aggressive tumor phenotypes (25–27). Notably, VANGL1 expression in endothelial cells further amplifies pro−angiogenic networks, indicating that VANGL1−driven vascular remodeling operates through both cell−autonomous and non−autonomous mechanisms (28). The recurrence of these patterns across diverse cancers suggests that VANGL1−mediated microenvironmental regulation represents a shared principle in tumor biology rather than a tissue−specific phenomenon.

Mechanistically, our bioinformatic and experimental data position VANGL1 at the nexus of polarity signaling and oncogenic pathways. The enrichment of Wnt, Notch, and mTOR pathways in VANGL1-associated gene sets, combined with strong correlations between VANGL1 expression and cancer stemness indices, provides a molecular rationale for the observed functional plasticity (29, 30). The planar cell polarity pathway, traditionally associated with tissue morphogenesis, appears coopted in malignancy to sustain stem cell self-renewal and invasive behavior (30). This aligns with emerging evidence that developmental signaling pathways are repurposed during tumorigenesis to maintain cellular plasticity and therapy resistance.

Our *in vitro* and *in vivo* studies demonstrated that VANGL1 functions as a driver that promotes cell proliferation, motility, and the maintenance of cancer stem cell properties. The pronounced tumor growth inhibition following VANGL1 depletion in hepatocellular carcinoma stem cell-derived xenografts underscores the therapeutic potential of targeting VANGL1 in stem cell-enriched tumors. Concurrently, VANGL1 knockdown also attenuated stromal activation. Our *in vivo* data demonstrated that VANGL1 inhibition led to the regression of dilated vascular lacunae and a reduction in vessel caliber. This phenotype is reminiscent of vascular normalization (structural pruning). While functional assays (e.g., perfusion or hypoxia measurements) are needed to confirm hemodynamic improvements, the structural transformation observed here strongly suggests that VANGL1 contributes to the maintenance of an aberrant, immature vascular network (31, 32).

This study has several limitations that should be acknowledged. First, although our analysis encompassed multiple cancer types, the depth of functional validation varied across different tumor contexts, with hepatocellular carcinoma receiving the most comprehensive experimental characterization; further validation with clinical samples is warranted. Second, while our TMA data demonstrate significantly elevated VANGL1 protein expression across multiple cancer types, direct correlation between VANGL1 expression and microvessel density via CD31 co-staining was not performed due to limited remaining tissue availability, and warrants future investigation. Third, although we added a Matrigel-based vascular-like tube formation assay using A1 hepatocellular carcinoma stem-like cells, this assay does not replace a standard HUVEC tube formation assay using conditioned medium from VANGL1-manipulated tumor cells. The current assay mainly supports a role for VANGL1 in tumor cell-intrinsic vascular-like network formation and cancer stem cell-associated plasticity. Future studies should employ endothelial-cell-based assays, including HUVEC conditioned-medium tube formation, endothelial migration, and permeability assays, to directly define the paracrine effects of VANGL1-expressing tumor cells on endothelial angiogenic behavior. Fourth, the *in vivo* validation was conducted in immunodeficient NCG mice to focus on human tumor growth and structural remodeling; future studies using syngeneic immunocompetent models are needed to functionally verify the recruitment of CD8^+^ T cells following VANGL1-mediated vascular normalization. Lastly, the subcutaneous xenograft model used for *in vivo* validation does not fully recapitulate the native hepatic microenvironment; future orthotopic implantation models are warranted to more faithfully evaluate liver-specific TME remodeling.

## Data Availability

The datasets used and analyzed in this study are all publicly available. Bulk transcriptomic and clinical data were obtained from The Cancer Genome Atlas (TCGA) and the Genotype-Tissue Expression (GTEx) database. Single-cell RNA sequencing data were obtained from the following public repositories: STAD (GEO: GSE167297), LIHC (GEO: GSE166635), NSCLC (ArrayExpress: E-MTAB-6149), OV (ArrayExpress: E-MTAB-8107), and CRC (GEO: GSE166555). Spatial transcriptomic data were obtained from the following sources: CRC (10x Genomics dataset repository: https://www.10xgenomics.com/datasets/human-colorectal-cancer-whole-transcriptome-analysis-1-standard-1-2-0), LIHC (Mendeley Data: skrx2fz79n), LUAD (GEO: GSE179572, sample GSM5420751), and OV (GEO: GSE211956, sample GSM6506115). All other data supporting the findings of this study are available from the corresponding author upon reasonable request.

## Supplementary material

Supplementary Figure 1 Pan-cancer expression landscape and diagnostic performance of VANGL1.

Supplementary Figure 2 Multi-omics correlates of VANGL1 dysregulation.

Supplementary Figure 3 Pan-cancer correlation between VANGL1 expression and cancer hallmark pathways.

Supplementary Figure 4 Validation of VANGL1 knockdown efficiency and quantification of sphere formation assays.

Supplementary Figure 5 VANGL1 co-expression network and functional module analysis in hepatocellular carcinoma.

## References

[1] Bejarano L Jordāo MJC Joyce JA . Therapeutic targeting of the tumor microenvironment. Cancer Discov. (2021) 11:933–59. doi: 10.1158/2159-8290.cd-20-1808. PMID: 33811125

[2] Weber F Reese KL Pantel K Smit DJ . Cancer-associated fibroblasts as a potential novel liquid biopsy marker in cancer patients. J Exp Clin Cancer Res. (2025) 44:127. doi: 10.1186/s13046-025-03387-7. PMID: 40259388 PMC12010557

[3] Lopes CDH Braganca Xavier C Torrado C Veneziani AC Megid TBC . A comprehensive exploration of agents targeting tumor microenvironment: Challenges and future perspectives. J Immunother Precis Oncol. (2024) 7:283–99. doi: 10.36401/jipo-24-23. PMID: 39524466 PMC11541921

[4] Wu P Gao W Su M Nice EC Zhang W Lin J . Adaptive mechanisms of tumor therapy resistance driven by tumor microenvironment. Front Cell Dev Biol. (2021) 9:641469. doi: 10.3389/fcell.2021.641469. PMID: 33732706 PMC7957022

[5] Guo Q Zhou Y Xie T Yuan Y Li H Shi W . Tumor microenvironment of cancer stem cells: Perspectives on cancer stem cell targeting. Genes Dis. (2024) 11:101043. doi: 10.1016/j.gendis.2023.05.024. PMID: 38292177 PMC10825311

[6] Li YR Fang Y Lyu Z Zhu Y Yang L . Exploring the dynamic interplay between cancer stem cells and the tumor microenvironment: Implications for novel therapeutic strategies. J Transl Med. (2023) 21:686. doi: 10.1186/s12967-023-04575-9. PMID: 37784157 PMC10546755

[7] Haibe Y Kreidieh M El Hajj H Khalifeh I Mukherji D Temraz S . Resistance mechanisms to anti-angiogenic therapies in cancer. Front Oncol. (2020) 10:221. doi: 10.3389/fonc.2020.00221. PMID: 32175278 PMC7056882

[8] Yang S Fang Y Ma Y Wang F Wang Y Jia J . Angiogenesis and targeted therapy in the tumour microenvironment: From basic to clinical practice. Clin Transl Med. (2025) 15:e70313. doi: 10.1002/ctm2.70313. PMID: 40268524 PMC12017902

[9] Yin Y Feng W Chen J Chen X Wang G Wang S . Immunosuppressive tumor microenvironment in the progression, metastasis, and therapy of hepatocellular carcinoma: From bench to bedside. Exp Hematol Oncol. (2024) 13:72. doi: 10.1186/s40164-024-00539-x. PMID: 39085965 PMC11292955

[10] Johnson A Townsend M O'Neill K . Tumor microenvironment immunosuppression: A roadblock to car t-cell advancement in solid tumors. Cells. (2022) 22:3626–0. doi: 10.3390/cells11223626 PMC9688327 36429054

[11] Dreyer CA VanderVorst K Carraway KL . Vangl as a master scaffold for wnt/planar cell polarity signaling in development and disease. Front Cell Dev Biol. (2022) 10:887100. doi: 10.3389/fcell.2022.887100. PMID: 35646914 PMC9130715

[12] Feng X Ye Y Zhang J Zhang Y Zhao S Mak JCW . Core planar cell polarity genes vangl1 and vangl2 in predisposition to congenital vertebral malformations. Proc Natl Acad Sci USA. (2024) 121:e2310283121. doi: 10.1073/pnas.2310283121. PMID: 38669183 PMC11067467

[13] Zhang K Yao E Chuang E Chen B Chuang EY Volk RF . Wnt5a-vangl1/2 signaling regulates the position and direction of lung branching through the cytoskeleton and focal adhesions. PloS Biol. (2022) 20:e3001759. doi: 10.1371/journal.pbio.3001759. PMID: 36026468 PMC9469998

[14] Huang J Luo S Shen J Lee M Chen R Ma S . Cellular polarity pilots breast cancer progression and immunosuppression. Oncogene. (2025) 44:783–93. doi: 10.1038/s41388-025-03324-0. PMID: 40057606 PMC11913746

[15] Dreyer CA VanderVorst K Natwick D Bell G Sood P Hernandez M . A complex of wnt/planar cell polarity signaling components vangl1 and fzd7 drives glioblastoma multiforme Malignant properties. Cancer Lett. (2023) 567:216280. doi: 10.1016/j.canlet.2023.216280. PMID: 37336284 PMC10582999

[16] VanderVorst K Dreyer CA Hatakeyama J Bell GRR Learn JA Berg AL . Vangl-dependent wnt/planar cell polarity signaling mediates collective breast carcinoma motility and distant metastasis. Breast Cancer Res. (2023) 25:52. doi: 10.1186/s13058-023-01651-2. PMID: 37147680 PMC10163820

[17] Peyravian N Nobili S Pezeshkian Z Olfatifar M Moradi A Baghaei K . Increased expression of vangl1 is predictive of lymph node metastasis in colorectal cancer: Results from a 20-gene expression signature. J Pers Med. (2021) 2:126. doi: 10.3390/jpm11020126. PMID: 33672900 PMC7918343

[18] VanderVorst K Hatakeyama J Berg A Lee H Carraway KL . Cellular and molecular mechanisms underlying planar cell polarity pathway contributions to cancer Malignancy. Semin Cell Dev Biol. (2018) 81:78–87. doi: 10.1016/j.semcdb.2017.09.026. PMID: 29107170 PMC5934344

[19] Knol JC Lyu M Böttger F Nunes Monteiro M Pham TV Rolfs F . The pan-cancer proteome atlas, a mass spectrometry-based landscape for discovering tumor biology, biomarkers, and therapeutic targets. Cancer Cell. (2025) 43:1328–1346.e8. doi: 10.1016/j.ccell.2025.05.003. PMID: 40446800

[20] Baghban R Roshangar L Jahanban-Esfahlan R Seidi K Ebrahimi-Kalan A Jaymand M . Tumor microenvironment complexity and therapeutic implications at a glance. Cell Commun Signal. (2020) 18:59. doi: 10.1186/s12964-020-0530-4. PMID: 32264958 PMC7140346

[21] Quail DF Joyce JA . Microenvironmental regulation of tumor progression and metastasis. Nat Med. (2013) 19:1423–37. doi: 10.1038/nm.3394. PMID: 24202395 PMC3954707

[22] Hao CC Xu CY Zhao XY Luo JN Wang G Zhao LH . Up-regulation of vangl1 by igf2bps and mir-29b-3p attenuates the detrimental effect of irradiation on lung adenocarcinoma. J Exp Clin Cancer Res. (2020) 39:256. doi: 10.1186/s13046-020-01772-y. PMID: 33228740 PMC7687693

[23] Cetin GO Toylu A Atabey N Sercan Z Sakizli M . Downregulation of vangl1 inhibits cellular invasion rather than cell motility in hepatocellular carcinoma cells without stimulation. Genet Test Mol Biomarkers. (2015) 19:283–7. doi: 10.1089/gtmb.2015.0014. PMID: 25874746 PMC4487596

[24] Park SY Kim H Yoon S Bae JA Choi SY Jung YD . Kitenin-targeting microrna-124 suppresses colorectal cancer cell motility and tumorigenesis. Mol Ther. (2014) 22:1653–64. doi: 10.1038/mt.2014.105. PMID: 24909917 PMC4435482

[25] Swanton C Bernard E Abbosh C André F Auwerx J Balmain A . Embracing cancer complexity: Hallmarks of systemic disease. Cell. (2024) 187:1589–616. doi: 10.1016/j.cell.2024.02.009. PMID: 38552609 PMC12077170

[26] Yan L Wu M Wang T Yuan H Zhang X Zhang H . Breast cancer stem cells secrete mif to mediate tumor metabolic reprogramming that drives immune evasion. Cancer Res. (2024) 84:1270–85. doi: 10.1158/0008-5472.can-23-2390. PMID: 38335272

[27] Ghalehbandi S Yuzugulen J Pranjol MZI Pourgholami MH . The role of vegf in cancer-induced angiogenesis and research progress of drugs targeting vegf. Eur J Pharmacol. (2023) 949:175586. doi: 10.1016/j.ejphar.2023.175586. PMID: 36906141

[28] Hatakeyama J Wald JH Printsev I Ho HY Carraway KL . Vangl1 and vangl2: Planar cell polarity components with a developing role in cancer. Endocr Relat Cancer. (2014) 21:R345–56. doi: 10.1530/erc-14-0141. PMID: 24981109 PMC4332879

[29] Galamb O Kalmár A Péterfia B Csabai I Bodor A Ribli D . Aberrant dna methylation of wnt pathway genes in the development and progression of cimp-negative colorectal cancer. Epigenetics. (2016) 11:588–602. doi: 10.1080/15592294.2016.1190894. PMID: 27245242 PMC4990228

[30] Dietrich B Haider S Meinhardt G Pollheimer J Knöfler M . Wnt and notch signaling in human trophoblast development and differentiation. Cell Mol Life Sci. (2022) 79:292. doi: 10.1007/s00018-022-04285-3. PMID: 35562545 PMC9106601

[31] Sahai E Astsaturov I Cukierman E DeNardo DG Egeblad M Evans RM . A framework for advancing our understanding of cancer-associated fibroblasts. Nat Rev Cancer. (2020) 20:174–86. doi: 10.1038/s41568-019-0238-1. PMID: 31980749 PMC7046529

[32] Shojaei F . Anti-angiogenesis therapy in cancer: Current challenges and future perspectives. Cancer Lett. (2012) 320:130–7. doi: 10.1016/j.canlet.2012.03.008. PMID: 22425960

