# Supplementary material for: VANGL1 links angiogenesis–stemness programs and tumor microenvironment remodeling: a pan-cancer, multi-omics study with translational validation
Source: Front Oncol. 2026 May 29;16:1836811. doi: 10.3389/fonc.2026.1836811 (PMC13259748; doi:10.3389/fonc.2026.1836811)
Supplement: Supplementary Figure 1 — Pan-cancer expression landscape and diagnostic performance of VANGL1. [file SupplementaryFile1.docx]

Supplementary Material

# Supplementary Figures

Systematic analysis of TCGA and GTEx databases revealed significant VANGL1 upregulation across 33 cancer types, with prominent overexpression in CESC, LUSC, BRCA, and STAD (Fig. S1A). VANGL1 demonstrated robust diagnostic performance for tumor versus normal discrimination (AUC > 0.7 in most cancers) (Fig.S1B), validated by independent GEO datasets (GSE116959, GSE14520, GSE39582, GSE26712, GSE29272) (Fig. S1C) and CPTAC proteomic cohorts (Fig.S1D), establishing VANGL1 as a conserved, highly expressed pan-cancer biomarker.


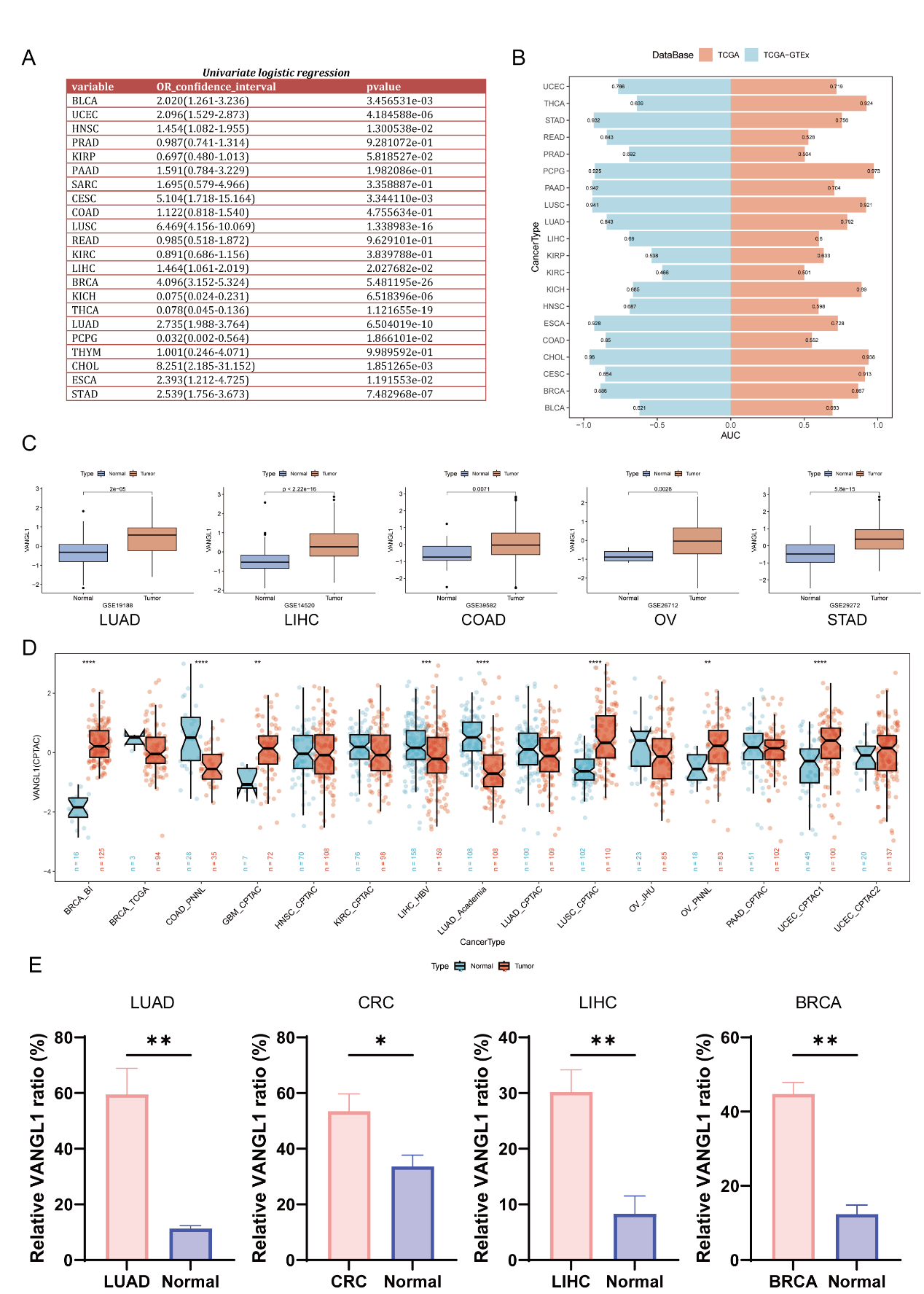


**Supplementary Figure 1.** Pan-cancer expression landscape and diagnostic performance of VANGL1. (A) Univariate logistic regression analysis of VANGL1 expression for tumor versus normal tissue classification across 33 cancer types. Odds ratios (OR) with 95% confidence intervals and p-values are shown. (B) Receiver operating characteristic (ROC) analysis comparing TCGA and GTEx databases. Bar plot displays area under the curve (AUC) values for VANGL1 in distinguishing tumor from normal tissues across cancer types. Red indicates TCGA data, blue indicates GTEx data. (C) Validation of VANGL1 upregulation in tumor versus normal tissues using independent GEO datasets. Box plots show VANGL1 expression in LUAD (GSE116959), LIHC (GSE14520), COAD (GSE39582), OV (GSE26712), and STAD (GSE29272). Statistical significance determined by Wilcoxon rank-sum test. (D) VANGL1 expression in paired tumor-normal samples from CPTAC and additional independent cohorts. (E) Relative VANGL1 expression ratio (%) quantified by QuPath image analysis in tumor and matched normal adjacent tissues across four cancer types (LUAD, CRC, LIHC, and BRCA). Data are presented as mean ± SD. *P < 0.05, **P < 0.01.

Across several tumors, VANGL1 mRNA correlated positively with GISTIC2.0 copy number (per-cancer Spearman ρ; Fig.S2A). Moderate correlations appeared in Rectum Adenocarcinoma (READ), Uveal Melanoma (UVM), LUSC, and LUAD. BH-FDR q values are shown where available; otherwise nominal P values. Probe-level analyses revealed inverse relationships between CpG β values at the VANGL1 locus and its mRNA in multiple cancers (e.g., LGG/GBM, BRCA, LUSC, LUAD), with a subset reaching FDR significance (Fig. S2B). These data are consistent with promoter-proximal hypomethylation accompanying higher expression; causality is not inferred. VANGL1 co-varied with m¹A/m⁵C/m⁶A regulators across cancers, typically with low-to-moderate |ρ| (Fig. S2C). The most consistent signals involved m⁶A machinery (METTL3/METTL14 writers, FTO eraser, YTHDF1/2/3 readers). Platform-provided BH-FDR values are displayed where available. Taken together, copy-number gain, locus-level hypomethylation, and coordinated variation with RNA-modification regulators support a multilayer model of VANGL1 regulation without asserting directionality.


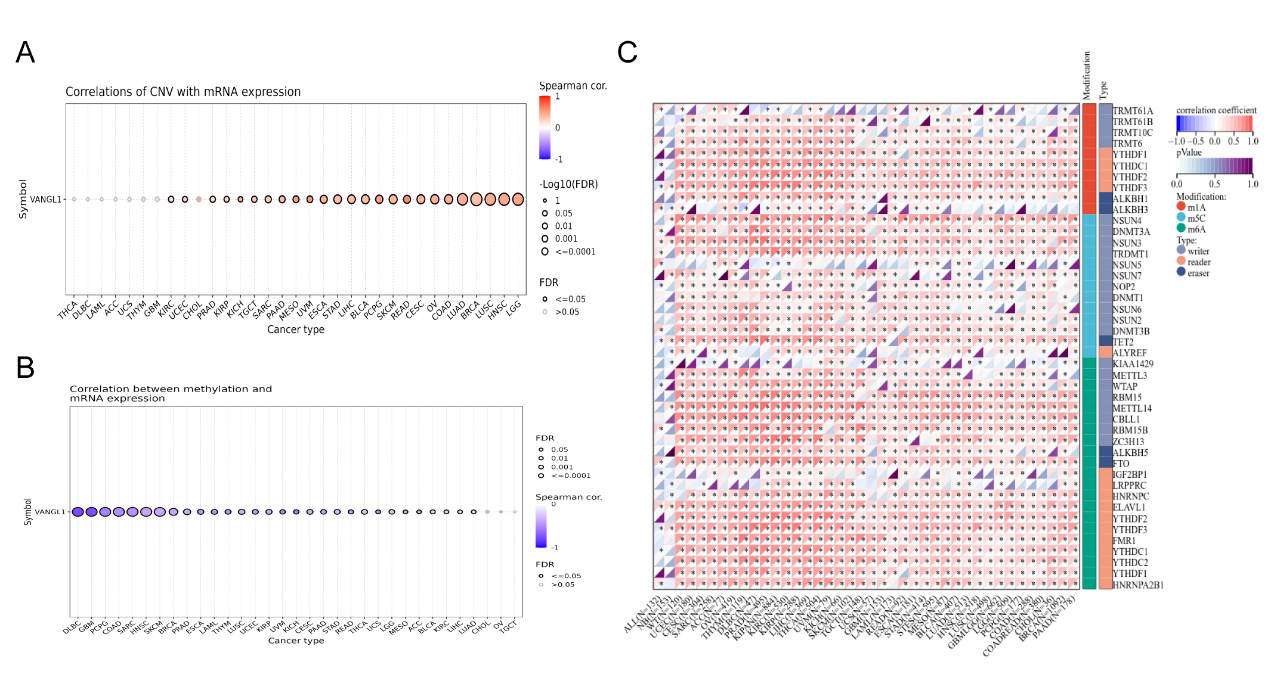


**Supplementary Figure 2.** Multi-omics correlates of VANGL1 dysregulation. (A) Spearman correlations between GISTIC2.0 copy number and VANGL1 mRNA (GSCA). Circle size denotes significance; color denotes direction/magnitude; q shown when available. (B) Strongest inverse correlation per cancer between VANGL1 mRNA and promoter-proximal CpG β (450K; TSS ±2 kb); q or P per portal. (C) Correlations between VANGL1 and 44 RNA-modification regulators (m¹A/m⁵C/m⁶A) in primary tumors (SangerBox). Heatmap shows coefficients (red positive, blue negative); class annotated on the right; intensity reflects −log₁₀(P) or −log₁₀(q).

To explore the functional implications of VANGL1 across malignancies, we examined its correlation with established cancer hallmark pathways using gene set variation analysis (GSVA). VANGL1 expression showed significant positive associations with metastasis and invasion signatures in LIHC, LUAD, and OV (Fig.S3A-B), consistent with its role in promoting tumor dissemination. Cell cycle signatures positively correlated with VANGL1 in multiple tumor types (Fig.S3C), supporting its proliferative functions observed in vitro. Notably, VANGL1 exhibited positive correlations with apoptosis pathways in several cancers (Fig.S3D). These pan-cancer analyses extend our single-cancer observations and establish VANGL1 as a broadly conserved regulator of malignant phenotypes.


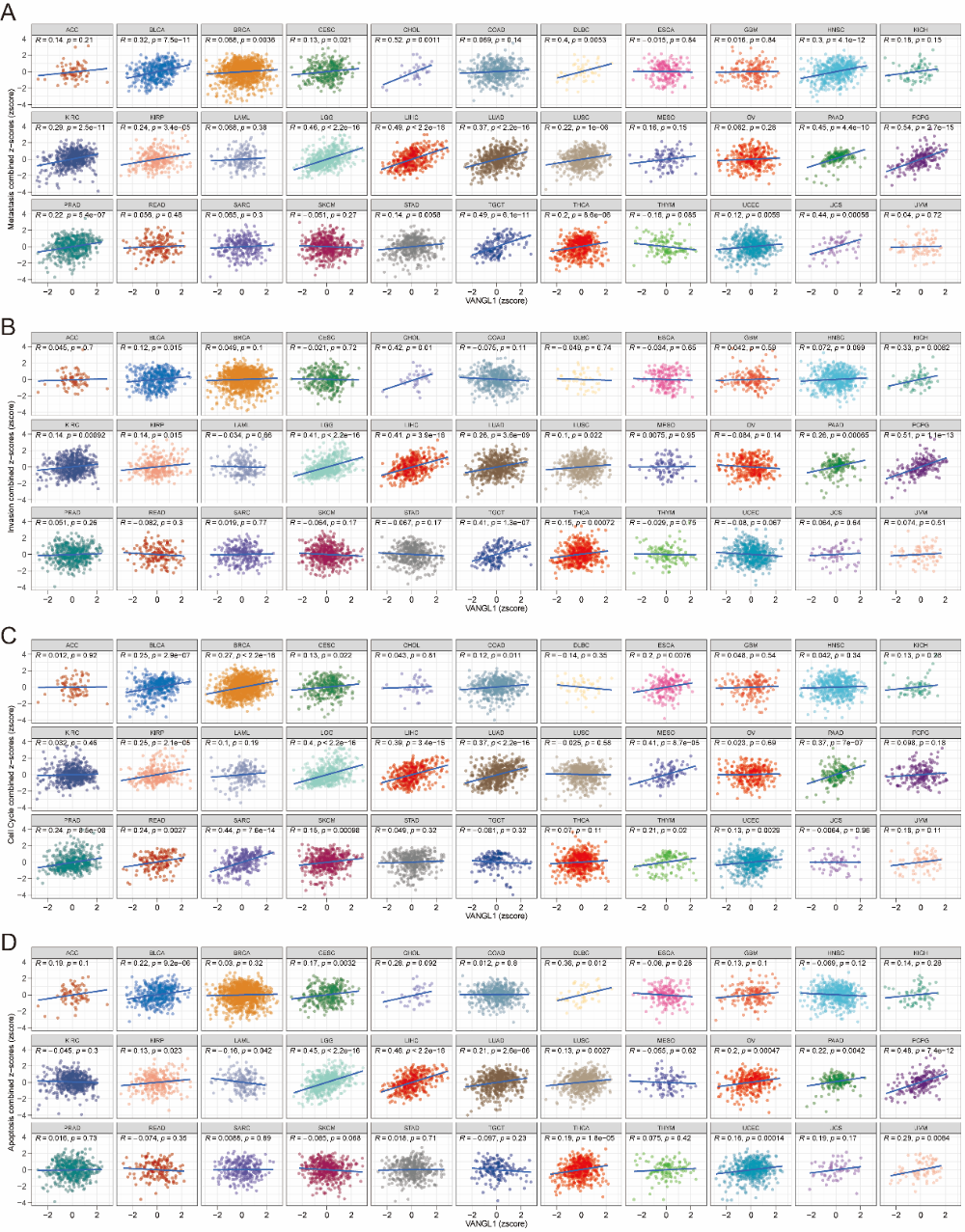


**Supplementary Figure 3.** Pan-cancer correlation between VANGL1 expression and cancer hallmark pathways. (A) Correlation analysis between VANGL1 expression and metastasis-associated gene signatures across 33 cancer types. Scatter plots display Pearson correlation coefficients (R) and p-values. Significant positive correlations observed in LIHC, LUAD, LUSC, and STAD indicate VANGL1's association with metastatic potential. (B) Correlation between VANGL1 expression and invasion-related gene signatures. Analysis reveals significant positive associations in multiple malignancies including BRCA, COAD, LIHC, LUAD, and OV, supporting VANGL1's role in tumor invasiveness. (C) Correlation between VANGL1 expression and cell cycle progression signatures. Strong positive correlations in BRCA, KIRC, LIHC, LUAD, and STAD suggest VANGL1 promotes proliferative capacity across diverse cancer types. (D) Correlation between VANGL1 expression and apoptosis-related signatures. Notably, VANGL1 shows positive correlations with apoptosis pathways in multiple cancers, including THCA, STAD, and UCEC, suggesting potential context-dependent roles in regulating programmed cell death.


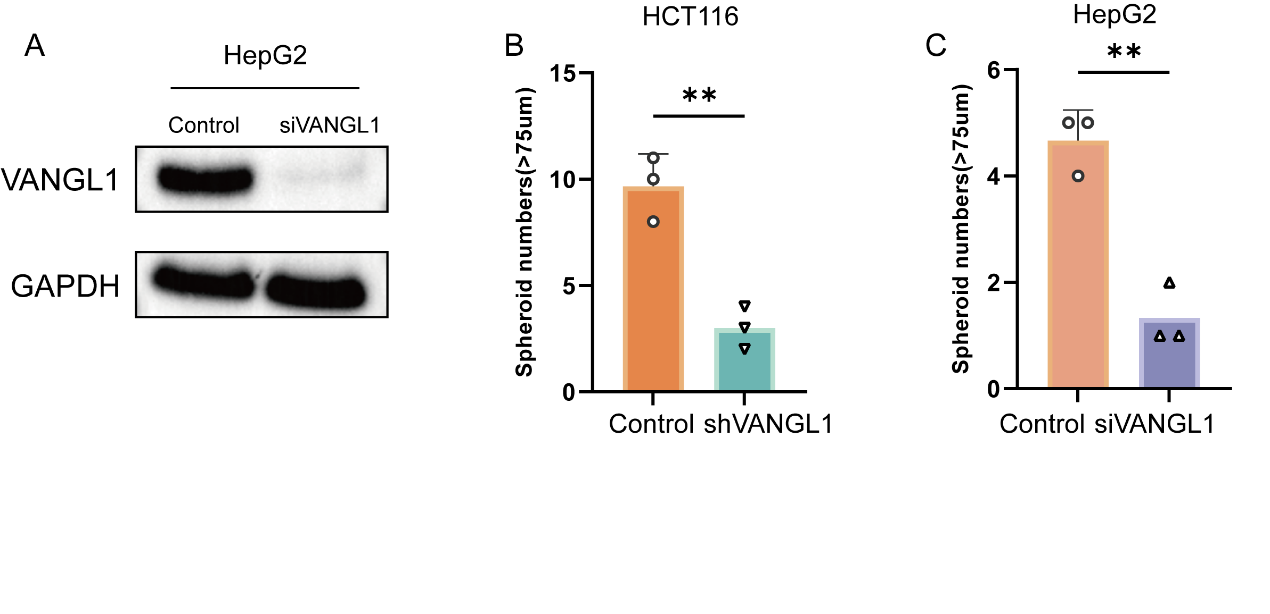


Supplementary Figure 4. Validation of VANGL1 knockdown efficiency and quantification of sphere formation assays. (A) Representative western blot showing VANGL1 protein expression in HepG2 cells transfected with siVANGL1 or negative control (siNC), confirming efficient knockdown of VANGL1 at the protein level. (B) Quantification of sphere number and sphere diameter in HCT116 cells stably expressing shVANGL1 or control construct (con), corresponding to Figure 7H. (C) Quantification of sphere number and sphere diameter in HepG2 cells transfected with siVANGL1 or siNC, corresponding to Figure 7I. Data are presented as mean ± SD from three independent experiments. *P< 0.05, **P < 0.01.

To systematically identify VANGL1-associated functional networks in hepatocellular carcinoma, we constructed gene co-expression matrices using TCGA-LIHC dataset. VANGL1 demonstrated robust co-expression with cell cycle regulators (TOP2A, CDK1, CCNB1), DNA repair components (BRCA1, RAD51), and chromosome segregation machinery (CDC20, PLK1) (Fig.S5A). Functional annotation of VANGL1-upregulated co-expression networks revealed enrichment in cell division, DNA repair, and viral carcinogenesis pathways (Fig.S5B-C), whereas downregulated networks were associated with thyroid hormone signaling and PPAR signaling (Fig.S5D-E), suggesting VANGL1-driven metabolic reprogramming. WGCNA further identified the blue module, characterized by cell cycle and DNA replication genes, as most strongly correlated with tumor grade and poor survival (Fig.S5F-G). Module pathway analysis confirmed TGF-beta signaling and viral carcinogenesis as key VANGL1-associated processes (Fig.S5H-I), mechanistically linking VANGL1 to both proliferative and microenvironmental remodeling programs in LIHC.


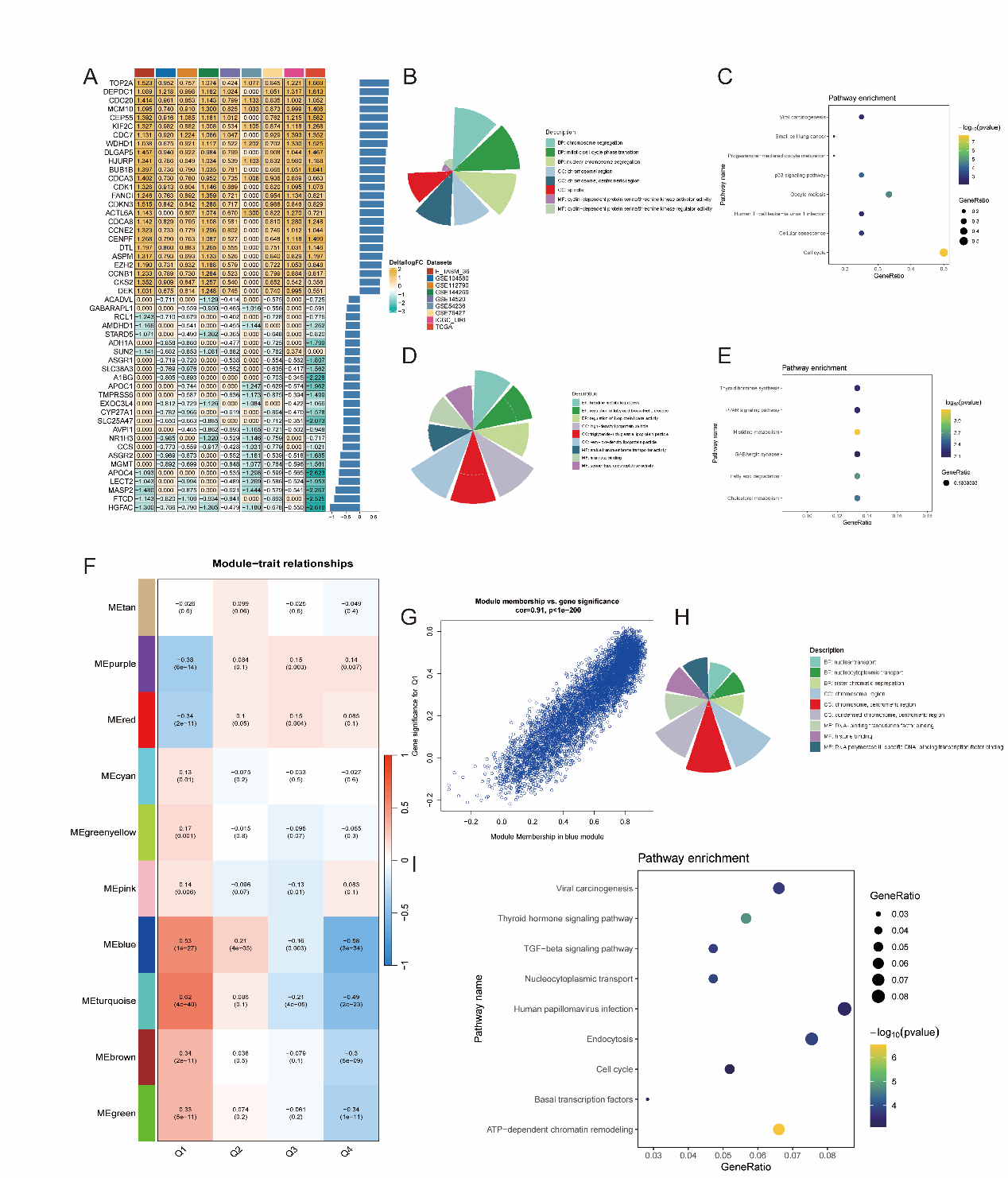


**Supplementary Figure 5.** VANGL1 co-expression network and functional module analysis in hepatocellular carcinoma. (A) Heatmap of top 50 genes co-expressed with VANGL1 in LIHC (TCGA-LIHC dataset). Color intensity indicates Pearson correlation coefficients; right panel shows cumulative correlation scores. VANGL1 demonstrates strong co-expression with cell cycle regulators (TOP2A, CDK1, CCNB1), DNA repair components (BRCA1, RAD51), and chromosome segregation machinery (CDC20, PLK1). (B) GO enrichment analysis of VANGL1 co-expression upregulated genes. Pie chart displays distribution of enriched biological processes including cell division, DNA repair, and chromosome segregation. (C) KEGG pathway enrichment of VANGL1 co-expression upregulated genes. Dot plot shows top enriched pathways ranked by GeneRatio and -log10(p-value), with viral carcinogenesis and small cell lung cancer as significant hits. (D) GO enrichment analysis of VANGL1 co-expression downregulated genes. (E) KEGG pathway enrichment of VANGL1 co-expression downregulated genes. (F) WGCNA module-trait relationships. Heatmap displays correlation coefficients between identified modules and clinical traits (tumor grade, stage, survival). Blue and brown modules show strongest associations with malignancy-related traits. (G) Scatter plot of module membership versus gene significance for the blue module, demonstrating high correlation (cor=0.91, p<1e-200) and robust module preservation. (H) GO enrichment of blue module genes. Pie chart shows predominant enrichment in cell cycle, DNA replication, and chromosome organization processes. (I) KEGG pathway enrichment of blue module. Dot plot highlights significant pathways including viral carcinogenesis, TGF-beta signaling, and cell cycle regulation.
